# Multiregion ultra‐deep sequencing reveals early intermixing and variable levels of intratumoral heterogeneity in colorectal cancer

**DOI:** 10.1002/1878-0261.12012

**Published:** 2016-10-20

**Authors:** Yuka Suzuki, Sarah Boonhsi Ng, Clarinda Chua, Wei Qiang Leow, Jermain Chng, Shi Yang Liu, Kalpana Ramnarayanan, Anna Gan, Dan Liang Ho, Rachel Ten, Yan Su, Alexandar Lezhava, Jiunn Herng Lai, Dennis Koh, Kiat Hon Lim, Patrick Tan, Steven G. Rozen, Iain Beehuat Tan

**Affiliations:** ^1^ Centre for Computational Biology Duke‐NUS Medical School Singapore Singapore; ^2^ Program in Cancer and Stem Cell Biology Duke‐NUS Medical School Singapore Singapore; ^3^ Institute of Cellular and Molecular Biology Singapore Singapore; ^4^ Department of Medical Oncology National Cancer Centre Singapore Singapore; ^5^ Department of Pathology Singapore General Hospital Singapore; ^6^ Genome Institute of Singapore, A*STAR Singapore; ^7^ Department of Colorectal Surgery Singapore General Hospital Singapore; ^8^ Colorectal Practice Mount Elizabeth Medical Centre Singapore Singapore; ^9^ Cancer Science Institute National University of Singapore Singapore

**Keywords:** colorectal cancer, Copy Number Variation, gene expression, genetic heterogeneity

## Abstract

Intratumor heterogeneity (ITH) contributes to cancer progression and chemoresistance. We sought to comprehensively describe ITH of somatic mutations, copy number, and transcriptomic alterations involving clinically and biologically relevant gene pathways in colorectal cancer (CRC). We performed multiregion, high‐depth (384× on average) sequencing of 799 cancer‐associated genes in 24 spatially separated primary tumor and nonmalignant tissues from four treatment‐naïve CRC patients. We then used ultra‐deep sequencing (17 075× on average) to accurately verify the presence or absence of identified somatic mutations in each sector. We also digitally measured gene expression and copy number alterations using NanoString assays. We identified the subclonal point mutations and determined the mutational timing and phylogenetic relationships among spatially separated sectors of each tumor. Truncal mutations, those shared by all sectors in the tumor, affected the well‐described driver genes such as *APC, TP53,* and *KRAS*. With sequencing at 17 075×, we found that mutations first detected at a sequencing depth of 384× were in fact more widely shared among sectors than originally assessed. Interestingly, ultra‐deep sequencing also revealed some mutations that were present in all spatially dispersed sectors, but at subclonal levels. Ultra‐high‐depth validation sequencing, copy number analysis, and gene expression profiling provided a comprehensive and accurate genomic landscape of spatial heterogeneity in CRC. Ultra‐deep sequencing allowed more sensitive detection of somatic mutations and a more accurate assessment of ITH. By detecting the subclonal mutations with ultra‐deep sequencing, we traced the genomic histories of each tumor and the relative timing of mutational events. We found evidence of early mixing, in which the subclonal ancestral mutations intermixed across the sectors before the acquisition of subsequent nontruncal mutations. Our findings also indicate that different CRC patients display markedly variable ITH, suggesting that each patient's tumor possesses a unique genomic history and spatial organization.

**Abbreviations**
ASCAT: allele‐specific copy number analysis of tumors
CRC: colorectal cancer
GAGE: generally applicable gene‐set enrichment for pathway analysis
GATK: Genome Analysis Toolkit
ITH: intratumor heterogeneity
LOH: loss of heterogeneity
PCR: polymerase chain reaction
PHYLIP: PHYLogeny Inference Package
VAF: variant allele frequency

## 1 Introduction

Colorectal cancer (CRC) is the third most common cancer worldwide (Ferlay *et al*., 2014). Large‐scale collaborative sequencing projects such as The Cancer Genome Atlas (TCGA) have catalogued genetic alterations across patients with CRC (interpatient heterogeneity) (Cancer Genome Atlas Network, 2012). Heterogeneity can also exist in a single tumor, as subpopulations of cancer cells with distinct genomic alterations may exist across different regions (intratumor heterogeneity or ITH). Analysis of ITH allows the reconstruction of the phylogenetic tree of subpopulations within a tumor and provides insights into the timing and pervasiveness of the genomic events that contributed to cancer development. At the same time, ITH poses significant challenges to cancer therapy by limiting our ability to personalize therapy based on a single biopsy and by contributing to therapeutic escape, drug resistance, and metastasis. Understanding ITH in CRC thus has important biological and clinical implications.

Intratumor heterogeneity has recently been characterized by multiregion sequencing in several major cancer types, including cancers of the prostate, breast, kidney, brain, ovary, liver, and lung (Boutros *et al*., 2015; de Bruin *et al*., 2014; Friemel *et al*., 2015; Gerlinger *et al*., 2012, 2014; Hoogstraat *et al*., 2014; Liu *et al*., 2009; Martinez *et al*., 2013; Navin *et al*., 2010; Sottoriva *et al*., 2013; Yates *et al*., 2015; Zhang *et al*., 2014). These studies described a variety of different evolutionary branching patterns, including both ‘palm‐like’ patterns, in which most mutations in likely driver genes were present in all regions of the tumor, and more branched, ‘oak‐like’ patterns, with many mutations in likely driver genes found in only one or a few regions (Table 1).

**Table 1:** Sequencing depths employed by previous studies in intratumor heterogeneity (ITH) in several cancer types

Cancer types	Sequencing experiment types and depth	Sequencing depth experiment	References
Colorectal	Whole exome seq, 68.16×	68.16	Kim *et al*. (2015)
	Whole exome seq, 20× + targeted capture seq, 626.58×	626.58	Sottoriva *et al*. (2015)
	Whole exome seq, 97.8× + deep sequencing (depth not provided by study)	97.8	Uchi *et al*. (2016)
Rectal	Whole exome seq, 47× + targeted capture seq, 400×	400	Hardiman *et al*. (2016)
Prostate	Whole genome, 30–50× + Ultra‐deep amplicon seq, 500×	500	Boutros *et al*. (2015)
Breast	Targeted capture seq, 265×	265	Yates *et al*. (2015)
Lung	Whole exome, 277× + targeted capture seq, 863×	863	Zhang *et al*. (2014)
	Whole exome/genome, 54–107× + targeted capture seq (depth not provided by study)	54 to 107	de Bruin *et al*. (2014)
Kidney	Whole exome (70×) + Ultra‐deep amplicon seq, 400×	400	Gerlinger *et al*. (2014)

Summary of sequencing depths employed by previous studies on ITH in other cancers including prostate, breast, lung, and kidney and CRC. The sequencing depths refer to the depths of the sequencing experiments.

In CRC, early studies were based on low‐throughput sampling of a few loci, for example, by sequencing PCR products from a few selected sites (Baisse *et al*., 2001; Losi *et al*., 2005; Naxerova *et al*., 2014; Thirlwell *et al*., 2010), while recent studies have examined ITH by whole exome sequencing of multiple tumor regions (Hardiman *et al*., 2016; Kim *et al*., 2015; Sottoriva *et al*., 2015; Uchi *et al*., 2016). These exome studies reported varying levels of ITH and both ‘palm’‐ and ‘oak’‐like phylogenetic structures (Table 1). However, these exome‐based sequencing studies were performed at relatively shallow depths, which might fail to detect somatic mutations with low variant allele frequencies. This could also overestimate ITH, if mutations that were actually shared across the regions were not detected in some of them, because of a low proportion of malignant cells or because the mutations were subclonal in some regions (McGranahan *et al*., 2015). This could also underestimate ITH, if a private subclonal mutation were missed altogether.

To address the need for understanding ITH in CRC comprehensively and accurately, we used targeted deep next‐generation sequencing (mean coverage 384×) of 799 genes to characterize the genetic profiles of four CRCs. We analyzed five primary tumor sectors and a matched normal tissue from each patient. Next, we performed ultra‐high‐depth amplicon sequencing (17 075×) to assess the presence or absence of each alteration in the various sectors. We also performed NanoString gene expression profiling and NanoString cancer copy number analysis.

## 2 Materials and methods

### 2.1 Subjects, samples, and consent

Fresh frozen primary tumors and matched normal tissues were harvested at surgery. None of the patients had undergone any systemic treatment prior to surgery. Two patients were in stage IV with liver metastasis and were undergoing palliative resection of the primary tumors, and the other two were in stage I and stage IIIb, respectively. Table S1 provides further clinicopathologic details. A pathologist carefully selected five spatially separated, ~ 1‐cm^3^ sectors from each tumor mass and one from adjacent normal mucosa. The samples were snap‐frozen and stored in liquid nitrogen. The study protocols were approved by the Institutional Review Board of Singapore Health Services (IRB approval number: 2011/439/B and 2011/110/B). Written informed consent for the use of tissue specimens for research was obtained from all participants. Short reads from our study have been deposited in the European Genome‐phenome Archive (EGA) under the study accession ID EGAS00001001720.

### 2.2 Targeted deep multiregion sequencing of 799 cancer‐associated genes

We performed the deep targeted multiregion hybrid‐capture sequencing with a custom Agilent SureSelect panel that focused on exons of 799 cancer‐related genes, as described by Tan *et al*. (Tan *et al*., 2015). This panel was derived from a comprehensive literature and database survey and comprises genes biologically and clinically relevant to cancer. DNA was extracted from the samples using the QIAamp Blood and Cell Culture DNA Mini Kit (Qiagen) according to the manufacturer's instructions. The quality and yield of the DNA samples were assessed by Quant‐IT PicoGreen dsDNA Assay (Invitrogen Life Technologies). The DNA was sheared using a Covaris S2 (Covaris Inc) to a size distribution of 150–200 bp. Sequencing libraries were prepared, and sequencing was carried out on HiSeq 2000 sequencers, and reads were aligned to the reference human genome. Average sequencing depth was 384× (range: 254×–709×, Table S2).

We called variants with two algorithms: Genome Analysis Toolkit (GATK)‐based algorithm (McKenna *et al*., 2010) and MuTect (Cibulskis *et al*., 2013) as previously described (Tan *et al*., 2015). The variants were curated by manually inspecting the sequencing reads corresponding to each variant using the Integrated Genomics Viewer (IGV) (Thorvaldsdóttir *et al*., 2013). We used the CCDS (Pruitt *et al*., 2009a), RefSeq (Pruitt *et al*., 2009b), Ensembl (Flicek *et al*., 2010), and UCSC known genes (Hsu *et al*., 2006) databases to annotate transcripts and amino acid changes. Detected somatic mutations included nonsynonymous and synonymous single‐nucleotide substitutions and small insertions/deletions. We classified mutations as one of the ‘truncal’ (common to all sectors), ‘branched’ (shared among ≥ 2, but not all, sectors), and ‘private’ (unique to one sector). There was no *a priori* VAF threshold for calling mutations, but all somatic mutation VAFs called by both callers were ≥ 2.5%.

### 2.3 Ultra‐deep amplicon sequencing to validate somatic mutations and assess their presence across all sectors

Using ultra‐deep amplicon sequencing, we sought to validate all candidate somatic mutations in Patients 1, 2, and 4 and to validate 42 of the 261 candidate mutations in the hypermutated tumor of Patient 3. The purpose of this was to confirm the presence of these somatic mutations and also to sensitively test for their presence in every sector of the tumor. The 42 candidate nonsynonymous mutations selected from Patient 3 comprised 4 truncal variants, 15 of 70 nonsynonymous branched variants, and 5 or 6 private variants in each sector. Across all patients, we successfully designed the primers for amplicon sequencing for 123 of 127 mutations initially identified for validation (Table S3). Primer design was carried out by using primer3 software (http://primer3.ut.ee). Singleplex PCR was performed on 10 ng of DNA from every sector of the tumor harboring the mutation to be amplified, the matched normal tissue, and three HapMap controls, namely NA18537, NA18542, and NA18545 (International HapMap 3 Consortium *et al*., 2010). The HapMap controls were of Han Chinese ancestry and were included as additional negative controls to help distinguish low variant allele frequency (VAF) somatic mutations from background sequencing artifacts. Primer pairs for two variants failed testing during PCR, leaving 121 pairs for validation. In total, there were 605 PCR amplifications for tumor sectors, 121 amplifications for the matched normal tissues, and 363 amplifications of the HapMap controls. Samples were sequenced with 155‐bp single‐end reads on the MiSeq sequencer (Illumina). Sequence reads were processed by adapter trimming and aligned to the hs37d5 genome using BWA‐mem (Li, 2013). Pileups were generated for each somatic variant using the samtools (Li *et al*., 2009) mpileup command. Counts of variant and the total reads for each tumor sector were tabulated. Average sequencing depth was 17 075× (range: 6383×–35 693×, Table S5).

The following stringent filters were applied to candidate somatic mutations in the ultra‐deep amplicon sequencing data to calculate ITH: (a) We only considered the sites with ≥ 100× coverage of nucleotides with base qualities ≥ 30 in normal and all tumor sectors and (b) we only counted as true‐positive sites lacking evidence of the mutation in both the normal sample and HapMap controls. This assessment was based on the mean and standard deviation of variant reads in the normal sample and controls (*Z* score of all variant reads in the tumor sectors > 3) and on visual examination in IGV (Thorvaldsdóttir *et al*., 2013). Data for five variants did not meet the criterion (a), leaving 116 primer pairs for which we could assess the false‐positive and false‐negative rates in the initial targeted sequencing. In total, 580 mutation sites were assayed by ultra‐deep sequencing. There was no *a priori* VAF threshold for calling mutations in the ultra‐deep sequencing, but all somatic mutation VAFs validated by ultra‐deep sequencing were ≥ 0.07%.

### 2.4 Identification of copy number alterations from sequencing data

For each tumor sector (paired with the appropriate normal sample), we estimated the proportion of malignant cells and identified the broad genomic regions of copy number gain or loss by applying the allele‐specific copy number analysis of tumors (ascat) software (Van Loo *et al*., 2010) to the single‐nucleotide polymorphism allele fractions and the relative read depths extracted from the exome sequencing data.

### 2.5 Subclonality analysis

Using the estimates of VAFs from the ultra‐deep sequencing data, we determined which truncal or branched nonsynonymous somatic mutations were likely subclonal. Using the estimated malignant cell fraction and genomic copy number at the site of a given mutation (both obtained from ASCAT analysis), we estimated the minimum expected VAF of that mutation. We estimated that a mutation was clonal if the observed VAF was (a) similar to or greater the minimum expected VAF and (b) similar to that of other mutations of the same group (i.e., truncal or branched) in the same sector. Conversely, if the observed VAF was substantially lesser than expected based on these criteria, we concluded that the mutation was likely subclonal. Details for each mutation analyzed in this way are in Table S4.

### 2.6 Phylogenetic analysis

We used the ‘discrete‐characters Wagner parsimony’ method in phylogeny inference package (phylip) version 3.695 (Felsenstein, 2005) to generate phylogenetic trees with the matched nonmalignant tissue as the outgroup (input to phylip). The trees were drawn using the DrawTree tool under the phylip package.

### 2.7 Detection of copy genomic number alterations and mRNA profiling with NanoString

About 300 ng of purified genomic DNA was mixed with NanoString nCounter Cancer Copy Number Variation CodeSets (NanoString Technologies, Seattle, WA, USA). This assay digitally measures the genomic copy numbers of 87 important genes that are often amplified or deleted in cancer. We digested the DNA with *AluI* and then hybridized with the CodeSets according to the manufacturer's protocol. The resulting signals were analyzed with the ncounter digital analyzer software. CodeSets with average counts > 3 were used for further analysis.

The NanoString nCounter platform was used to quantify mRNA levels. About 1 μg of total RNA was extracted from 20 to 25 mg of frozen tissue and hybridized to the NanoString Pan Cancer Panel CodeSets. The nsolver software tabulated the raw counts, checked the quality, and normalized. Due to low malignant cell content, we excluded one sector from the analysis for Patient 3. We conducted a gene pathway analysis based on the log2‐transformed counts using the generally applicable gene‐set enrichment for pathway analysis (GAGE) R package (Luo *et al*., 2009). Pathway dysregulation scores and the associated *P*‐values were calculated for each of the 13 cancer pathways represented by the NanoString Pan Cancer Panel CodeSets, using the patient's nonmalignant tissue as the baseline reference. Pathways with adjusted *P*‐values < 0.1 were selected for further analysis. Hierarchical clustering and plotting based on the dysregulation scores of the pathways was carried using the heatmap.2 function in R using default parameters.

## 3 Results

### 3.1 Genetic ITH varies across tumors

**Figure 1:**
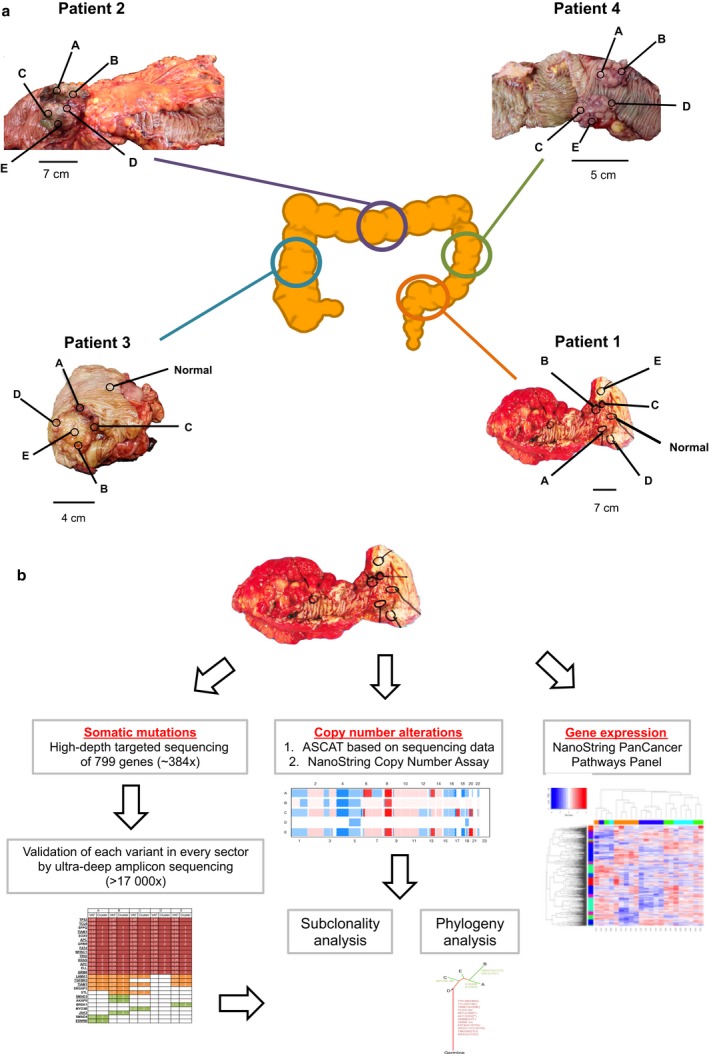
Locations of tumors and biopsies sampled and general study workflow. (a) Locations of tumors and tissue sectors. Multiple tumor Sectors A, B, C, D, and E, and a nonmalignant Sector N, were taken from the locations shown. (b) A combination of mutational profiling (by the targeted capture sequencing and ultra‐deep amplicon sequencing), copy number alteration profiling (by ASCAT and NanoString Copy Number Assay), and gene expression profiling (by NanoString Pan Cancer Pathways Panel Assay) was employed in this study.

We used deep targeted sequencing of 799 cancer‐associated genes in 24 spatially separated tissue sectors from four patients (five tumor sectors and one matched nonmalignant tissue per patient) to comprehensively catalogue somatic mutations in different regions of the tumors. Figure 1 summarizes the general workflow of our study.

We validated the mutations with ultra‐deep amplicon sequencing, which also detected the presence of some of the mutations at low VAFs in sectors in which they were not identified by the initial targeted deep sequencing. Ultra‐deep sequencing confirmed 289 of the 307 candidate somatic mutations detected in the initial targeted sequencing, for a true‐positive rate of 94% (289/307). Of the 18 false positives, 17 appeared to be germline variants that were seen in the adjacent normal tissue or HapMap controls. The remaining variant was not observed in any sample in the ultra‐deep sequencing. In addition, ultra‐deep sequencing detected 76 mutations that had been missed by the initial targeted sequencing, yielding a false‐negative rate of 21% (76/(289 + 76) in the original targeted sequencing. Of these, 72 were probably missed by the initial targeted sequencing because of low VAFs and four were missed due to low coverage of the targeted capture sequencing data in the tumor sector.

**Figure 2:**
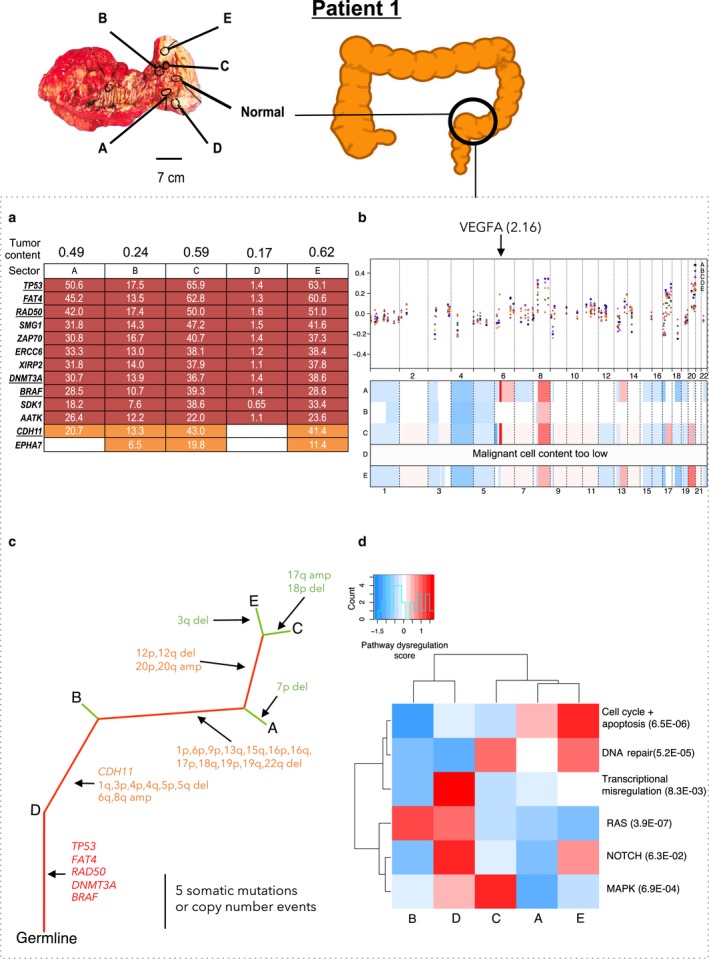
Patient 1 in stage IIIb with no localized metastasis. (a) Distribution of nonsynonymous (underlined) and synonymous mutations across sectors. VAF: variant allele frequency. Truncal mutations (those present in all sectors) are in red, branched mutations (nontruncal mutations shared by ≥ two sectors) are in orange, and private mutations (unique to one sector) are in green. (b) Copy number alterations in each sector of each tumor. First panel for each patient in which one dot for each sector shows the copy number ratio quantified for one of the 87 genes in the NanoString nCounter v2 Cancer CN Assay. The *x*‐axis indicates genomic position; the *y*‐axis indicates the normalized copy number ratio. The second panel for each sector is a heatmap showing ASCAT‐estimated copy numbers across the genome. White indicates copy number equal to the average ploidy of the sector; red indicates copy number gains; blue indicates copy number loss. Arrow indicates the location of the highly amplified *VEGFA* gene with the maximum copy number ratio of 2.16. Heatmap was plotted using R package CopyNumber. (c) Phylogenetic trees for the tumor sectors based on the detected mutations and copy number alterations. Color scheme for truncal, branched, and private mutations is in panel b. Nonsynonymous mutations and indels were indicated on the trunk and branches. (d) Significantly dysregulated pathways across the tumor sectors in each patient were identified using R package GAGE. Pathway scores and q‐values were calculated based on mRNA levels of the 800 genes in the NanoString Pan Cancer Pathways Panel Assay.

Patient 1 had stage IIIb rectosigmoid carcinoma without distant metastases (Fig. 2, Table S1). This carcinoma was highly homogeneous, with 85% of mutations being truncal, as indicated by the long trunk in Fig. 2. These truncal mutations affected the known CRC drivers, including *TP53, FAT4,* and *BRAF* (Hisamuddin and Yang, 2006). The only nonsilent branched mutation was in *CDH11*, which is not widely considered a driver in CRC. Therefore, we postulate that in this tumor, all the mutations required for tumorigenesis occurred before the last clonal expansion (Fig. 2). Unless the cells from the last clonal expansion actually replaced preexisting tumor cells, this suggests that these mutations occurred when the tumor was still small.

**Figure 3:**
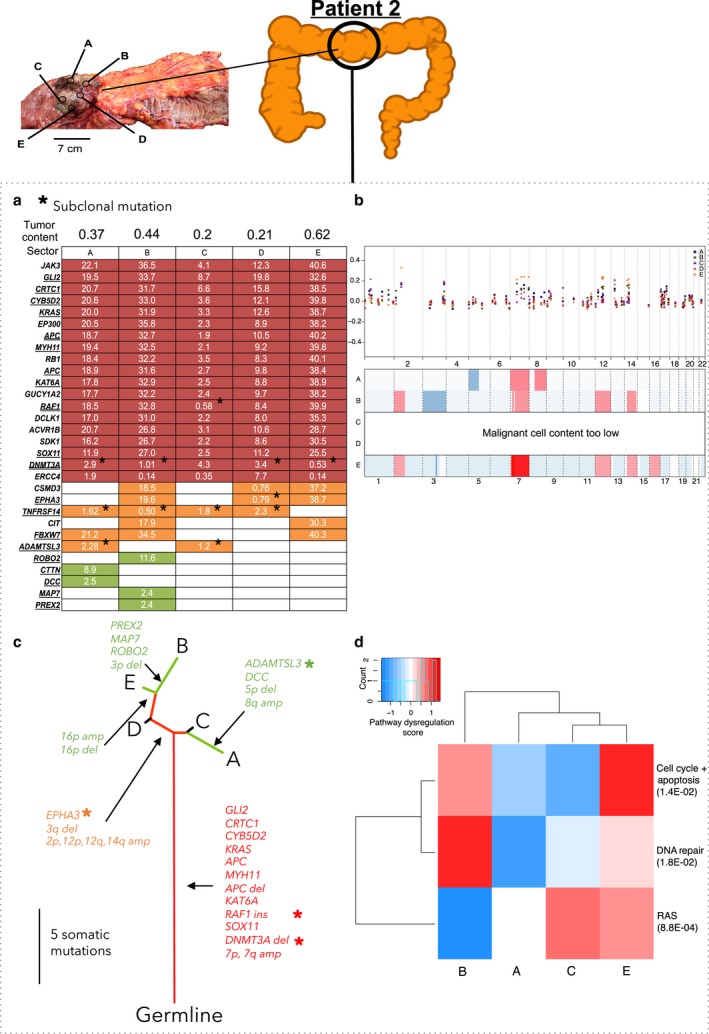
Patient 2 in stage IV with metastasis to the liver. Panels (a,b,c,d) as in Fig. 2. (b) Panel b as in Fig. 2, with the additions that double underline indicates microindel mutations and asterisks (*) indicate the subclonal nonsynonymous mutations.

Patient 2 had stage IV transverse colon carcinoma with liver‐limited metastasis (Fig. 3, Table S1). Of the mutations, 61% were truncal, including those in *KRAS* and *APC* (Fig. 3). A majority of these truncal mutations were likely clonal, except for an insertion in *RAF1* and a deletion in *DNMT3A* that were subclonal in one sector and four sectors, respectively (Fig. 3, Table S4). Interestingly, we also observed clonal mutations that were branched or private. This suggests the possibility that, despite being acquired later, these mutations became clonal because they conferred a selective advantage. For example, *FBXW7,* a known driver gene of CRC (Rajagopalan *et al*., 2004; Wood *et al*., 2007), was clonally mutated in Sectors A, B, and E.

**Figure 4:**
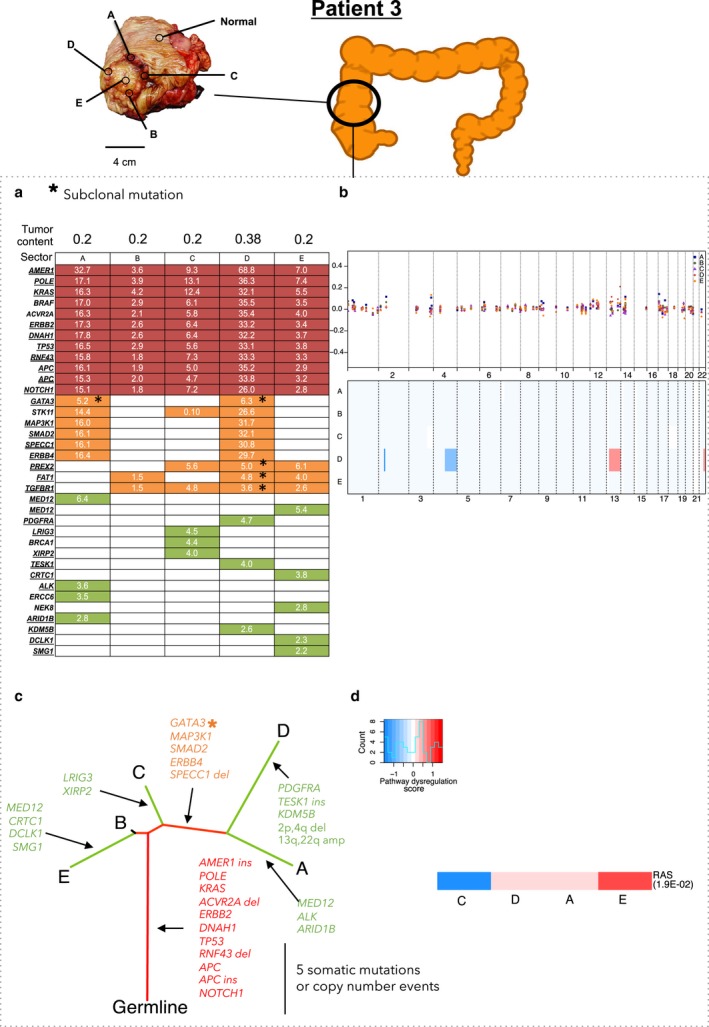
Patient 3 in stage IV with metastasis to the liver. This patient had a hypermutated tumor. Panels (a) through (d) as in Fig. 3.

Patient 3 had microsatellite unstable stage IV primary adenocarcinoma in the ascending colon and liver‐limited metastasis (Fig. 4, Table S1). The tumor was hypermutated (56.7 somatic mutations per Mb, Table 2), showed the mutation signature of mismatch repair deficiency (Alexandrov *et al*., 2013), and had MSH‐2 and MSH‐6 protein losses as determined by immunohistochemistry (Fig. 4, Tables 2 and S1). There was no evidence of somatic mutations in *MSH2* or *MSH6* in this patient. Microsatellite instability is often due to epigenetic silencing of *MLH1*. This tumor had the highest ITH: Only 33% of the mutations were truncal (Fig. 4, Table 2). The extensive heterogeneity stemmed from the high overall mutation rate (56.7 mutations per Mb).

**Table 2:** Summary of ITH in each tumor

Patients	Stages	MSI/MSS status	MMR deficiency signature	SNV and microindel mutation details and ITH					Degree of ITH	
				Overall mutation rate (mut per Mb)	Percentage of heterogenous mutations	Clonal mutations	Subclonal mutations	Evidence of early intermixing	Transcriptomic	Copy number
1	IIIb	MSS	No	4.13	15.4	*TP53, FAT4, RAD50, DNMT3A, BRAF*	None	No	Low	High
2	IV	MSS	No	9.35	36.7	*GLI2, CRTC1, CYB5D2, KRAS, APC, MYH11, APC* indel*, KAT6A, ACVR1B* indel*, SOX11*	*DNMT3A* indel^*(T)*^, *RAF1* indel^*(T)*^, *EPHA3, TNFRSF14, ADAMTSL3*	Yes	High	High
3	IV	*MSH2* and *MSH6* losses	Yes	56.7	66.7	*POLE, KRAS, ACVR2A* indel*, ERBB2, DNAH1, TP53, RNF43, APC, APC* indel*, NOTCH1*	*GATA3, PREX2* indel*, FAT1, TGFBR1* indel	No	High	Low
4	I	MSS	No	6.74	48	*TP53, TRIO, TIAM1, APC, FAT4, GRM8* indel, *SOX9*	*WHSC1* ^*(T)*^ *, GRM8* ^*(T)*^ *, LAMA1, TGFBR2, TIAM1*	Yes	Low	Intermediate

Summary of ITH in each tumor at the somatic mutational, transcriptomic, and copy number levels. Heterogeneous mutations refer to nontruncal mutations. Coding mutations that are clonal and subclonal are summarized in this table; ^*(T)*^ indicates truncal mutation; see also Figs 2 through 5.

**Figure 5:**
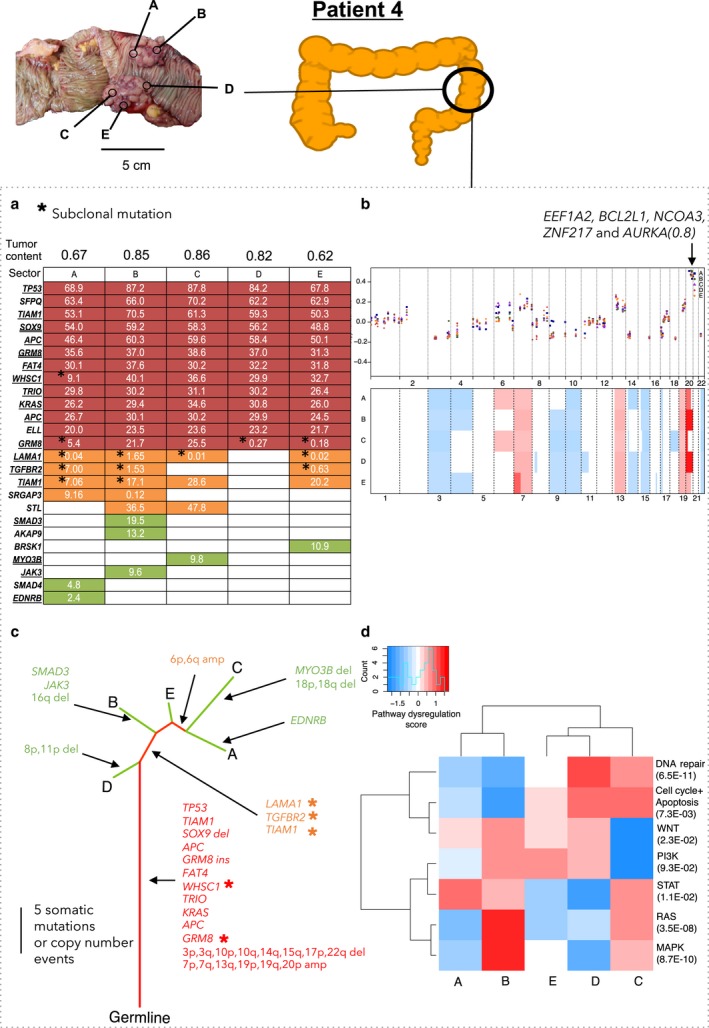
Patient 4 in stage I with no metastasis. Panels (a) through (d) as in Fig. 3. Arrow on panel (b) marks the amplified region containing the *EEF1A2, BCL2L1, NCOA3, ZNF217,* and *AURKA* genes, with the maximum copy number ratio of 0.8.

Patient 4 had stage I primary carcinoma in the descending colon (Fig. 5, Table S1). The tumor had high genetic ITH: Only 52% of mutations were truncal (Fig. 5, Table 2). We noted subclonal, nonsynonymous truncal mutations in *WHSC1* (subclonal in Sector A) and *GRM8* (subclonal in Sectors A, D, and E, Fig. 5, Table S4). There was also a subclonal, nonsynonymous branched mutation in *TIAM1*, which has been implicated in aggressiveness of CRC cells *in vivo* (Fig. 5, Table S4) (Malliri *et al*., 2006; Minard *et al*., 2005).

Differences in genetic ITH are likely contributed by the variation in mutation rates in the tumors. The hypermutated tumor with the highest mutation rate of 56.7 mutations per Mb exhibited significantly greater ITH than the rest (Table 2).

### 3.2 Copy number and mRNA profiles offered additional perspectives on ITH

Based on the targeted sequencing data, we identified the copy number alterations in a genomewide scale. We identified the losses in chromosome arms 3p and q, 4p and q, and 17p and gains in chromosome arms 6p, 7p, 7q, 13q, and 20p and q, all in at least two patients. These have been previously reported (Beroukhim *et al*., 2010; Cancer Genome Atlas Network, 2012; Zack *et al*., 2013) (Table S8). Copy number ITH was highest in Patient 2: There were 12 copy number alterations, of which only two were truncal (Fig. 3, Table S8). Copy number ITH was also high in Patient 1: Of 34 copy number alterations, only seven were truncal (Fig. 2, Table S8). Copy number ITH was lower, but still substantial, in Patient 4: Of 28 copy number alterations, 13 were truncal (Fig. 5, Table S8). Patient 3's tumor, consistent with the previous reports of genome stability in microsatellite unstable tumors (Cancer Genome Atlas Network, 2012), had a largely diploid genome with only two copy number alterations and thus very low copy number ITH (Fig. 4, Table S8).

NanoString nCounter Cancer analysis of copy number alterations at 87 genes that are commonly amplified or deleted in cancer was in general consistent with the analysis based on sequencing data and ASCAT. The NanoString data supported the general observation that the copy number alterations were largely similar across sectors (Figs 2, 3, 4, 5). Hierarchical clustering of dysregulated pathways appeared to mirror the phylogenetic relationships in Patient 1, but for Patients 2, 3, and 4, a robust relationship between dysregulated pathways and phylogeny was absent (Figs 2, 3, 4, 5). This is not surprising, as, in addition to reflecting the genetic heterogeneity of malignant cells *per se*, gene expression ITH likely also reflects the variation in the proportions of nonmalignant as well as malignant cell populations across sectors.

We studied the relationships between copy number ITH and gene expression for the 42 genes assayed by both the NanoString copy number and mRNA panels. Supporting the hypothesis that ITH in gene expression is sometimes driven by copy number ITH, in Patient 1, we identified three genes, *VEGFA, ITGB4,* and *GRB2,* with genomic amplification and overexpression only in Sectors A, C, and E (Figs 2 and S3). In addition, in Patient 4, there was a truncal genomic copy number gain associated with high expression in genes involved in the PI3K/Akt pathway (*AKT2, BCL2L1*, and *CCNE1*) and there was a truncal copy number loss associated with low expression of *PTEN* and *PIK3CA* (Figs 5 and S4).

## 4 Discussion

### 4.1 Targeted deep sequencing followed by ultra‐deep sequencing improved ITH analysis

The depth (384×) of the initial sequencing that focused on cancer‐associated genes ensured the sensitive initial detection of somatic mutations and made ultra‐deep amplicon sequencing of the identified somatic variants across every tumor sector manageable. With the targeted sequencing, we may miss diversity in regions of the genome that we did not target and whose functional significance is not currently known. However, the targeted sequencing detected adequate numbers of mutated sites to reconstruct tumor phylogenies across the sectors.

Subsequent ultra‐deep sequencing dramatically improved the precision of the analyses of ITH and tumor phylogenies. Ultra‐deep sequencing detected the mutations that had very low VAFs, either because of a low proportion of malignant cells, because the mutations were present only in subclones, because the mutations were present on single chromosomes in hyperploid genomic regions, or because of a low proportion of malignant cells in the tumor sample (Table S7). This was true for some tissue samples (Figs 2, 3, 4) with low tumor purity, which partly reflects the initially observed genetic ITH. However, validation by ultra‐deep sequencing further confirmed the presence of mutations that were missed by the initial sequencing. Thus, many mutations that appeared to be branched in the initial targeted sequencing data were revealed to be truncal by ultra‐deep sequencing (Table 3, Fig. S1). Similarly, many mutations that appeared to be private in the targeted sequencing data were revealed to be branched in the ultra‐deep sequencing data. Indeed, heterogeneity (defined as the proportion of nontruncal mutations in the tumor) estimated in the ultra‐deep sequencing was 4.0% to 38.4% lower than the heterogeneity estimated in the initial targeted sequencing data (Table 3). Ultra‐deep sequencing was also critical for distinguishing the subclonal from the clonal mutations. For example, in Patient 2, the targeted sequencing identified the canonical *KRAS* G12D mutation in four of five sectors; ultra‐deep sequencing showed that the mutation was truncal and clonal (Fig. S1). In summary, ultra‐deep sequencing was critical for the identification of somatic mutations that were present across all sectors of the tumor and for an accurate assessment of ITH.

**Table 3:** Comparison of intratumor heterogeneity (ITH) assessed by deep targeted sequencing and ultra‐deep amplicon sequencing

Patients	Targeted hybrid‐capture sequencing (~ 384×)			Ultra‐deep sequencing (~ 17 075×)			Reduction in heterogeneity after ultra‐deep sequencing (%)
	The number of mutations detected		Percentage of heterogeneous mutations	The number of mutations detected		Percentage of heterogeneous mutations	
	Heterogeneous	Total		Heterogeneous	Total		
1	7	13	53.8	2	13	15.4	38.4
2	20	30	66.7	11	30	36.7	30.0
3	31	36	86.1	24	36	66.7	19.4
4	13	25	52.0	12	25	48.0	4.0

Comparison of ITH mutational profiles estimated by deep targeted sequencing (~ 384×) and ultra‐deep amplicon sequencing (~ 17 075×) in four patients. The ITH profile is reflected by the percentage of heterogeneous mutations (i.e., mutations that are found in four or fewer tumor sectors).

Another technique that can detect the variants with very low VAFs is digital PCR (Day *et al*., 2013). However, this technique requires allele‐specific primers or probes for each variant (Vogelstein and Kinzler, 1999), which for some variants are difficult to design. Furthermore, each digital PCR reaction involves partitioning into hundreds or thousands of replicate reactions, which is infeasible (costly) even with current microfluidics or droplet methods (Huggett *et al*., 2015). By contrast, the ultra‐deep sequencing used in the present study required only a single sequencing run post‐PCR for all of the 24 sectors and still detected the variants with very low VAFs.

### 4.2 Evidence for early intermixing of truncal mutations

Multiregional sequencing studies provide insights into mutational timing, with the truncal mutations likely to have occurred earlier than the branched or private mutations. Interestingly, in Patients 2 and 4, some of these truncal mutations were subclonal in more than one sector, despite being likely early mutations (Figs 3 and 5). This suggests intermixing of subclones bearing different mutations during early stages of cancer development and could be consistent with the recently proposed ‘Big Bang model’ (Sottoriva *et al*., 2015). In this model, subclonal mutations appear early, producing a spatial mix of clones, and not only selection but also the timing of mutations contributes to ITH and the relative clonal compositions of various regions of a developing tumor. Although the sizes of our tumor sectors (1 cm^3^) may be too large to definitively support the Big Bang model, our observations highlight that some, but not all, CRCs exhibit this intermixing phenomenon. Alternatively, these observations may also be explained by the existence of neutral mutations that occur early and that, absent selection, remain at subclonal levels in an expanding malignancy (Uchi *et al*., 2016; Williams *et al*., 2016). Examples of subclonal truncal mutations include a small deletion in *DNMT3A* in Patient 2 and mutation in *GRM8* in Patient 4 (Table S4).

### 4.3 Varying levels of ITH across tumors and therapeutic implications

Our study demonstrated that the degree of ITH varies across different CRC tumors. This is important because ITH is thought to be a major determinant of a cancer's aggressiveness, resistance to therapy, and affects the patient's clinical course (McGranahan and Swanton, 2015). A different genomic history characterizes each cancer and in some but not all cancers; imprints of early intermixing are observed. These divergent genomic histories and ITH among CRC tumors point out that interpatient heterogeneity is not just in the complement of mutations but also the degree of intratumoral heterogeneity and genomic history within each patient, adding an additional layer to the concept of personalized medicine. We also note that sequencing to identify the potential therapeutic vulnerabilities based on one biopsy might be sufficient for some patients, but not for others. This highlights the need for large prospective studies with multiregional profiling and serial tissue sampling to understand how each patient's tumor evolves across space and time and how this translates to the patient's response to treatment. These studies may be critical for identifying potentially poor responders that share the same ITH profiles and developing better therapeutic interventions for them. Such studies are already ongoing in lung cancer (Jamal‐Hanjani *et al*., 2014) with other studies being planned for in different cancers including CRC.

## 5 Conclusions

This study provided a thorough assessment of ITH in CRC, made possible by deep targeted next‐generation sequencing followed by ultra‐deep amplicon sequencing, which reduced the false negatives and enabled the detection of subclonality. We observed the variable patterns of ITH, with the proportion of nontruncal mutations varying from 15% to 67%. The tumors also differed in that two had evidence of early mixing, in the form of subclonal, truncal mutations, while two did not. Tumor evolution and ITH will have an impact on tumor biology and clinical behavior. Larger prospective studies may determine how the patterns of ITH could be used to help predict disease progression and inform therapy selection.

## Author contributions

YS, IBT and SGR conceived and designed the study. KR and AG extracted DNA, prepared the libraries for next‐generation sequencing, and performed the Illumina HiSeq sequencing. HDL and JC designed the primers and performed the ultra‐deep amplicon sequencing validation. SBN performed the Illumina MiSeq sequencing. LSY analyzed the ultra‐deep amplicon sequencing data. SY and AL performed the NanoString Cancer copy number and NanoString Pan Cancer Panel CodeSets profiling. RT recorded clinical data. LWQ, LJH, DK and LKH provided the annotated clinical samples. SGR, PT, SBN, CC and IBT provided scientific input for the computational and biological analysis. YS, IBT and SGR interpreted all the data and wrote the manuscript. All authors read and approved the final manuscript.

## Supporting information


**Fig. S1.** Comparison of somatic mutations called by targeted hybrid‐capture sequencing and ultra‐deep amplicon sequencing.

**Fig. S2.** ASCAT profiles of twenty tumor sectors belonging to four patients.

**Fig. S3.** Correlations between genomic copy number and expression of *ITGB4, GRB2 and VEGFA*.

**Fig. S4.** Elevated copy number and higher expression of *AKT2, BCL2L1* and reduced copy number and lower expression of *PTEN* and *PIK3CA*.

**Table S1.** Summary of clinicopathologic and treatment details for the four patients.

**Table S2.** Summary of sequencing metrics of targeted hybrid‐capture sequencing.

**Table S3.** List of primer sequences used to validate mutations with ultra‐deep amplicon sequencing.

**Table S4.** List of somatic SNVs and indels that are subclonal (in red).

**Table S5.** Summary of sequencing metrics of ultra‐deep amplicon sequencing.

**Table S6.** Summary of somatic mutations validated by ultra‐deep amplicon sequencing and their variant allele frequencies.

**Table S7.** ASCAT‐estimated malignant cell content and average ploidy of each tumor sector.

**Table S8.** Amplified and deleted regions across four patients based on ASCAT data.
