# Supplementary material for: Multiregion ultra‐deep sequencing reveals early intermixing and variable levels of intratumoral heterogeneity in colorectal cancer
Source: Mol Oncol. 2016 Oct 20;11(2):124–39. doi: 10.1002/1878-0261.12012 (PMC5527459; doi:10.1002/1878-0261.12012)
Supplement: Supplementary file 1 — Fig. S1. Comparison of somatic mutations called by targeted hybrid‐capture sequencing and ultra‐deep amplicon sequencing. [file MOL2-11-124-s001.pdf]

Fig S1.

Patient 1

	A	B	C	D	E
TP53	0.506	0.175	0.659	0.014	0.631
FAT4	0.452	0.135	0.628	0.013	0.606
RAD50	0.420	0.174	0.500	0.016	0.510
SMG1	0.318	0.143	0.472	0.015	0.416
ZAP70	0.308	0.167	0.407	0.014	0.373
ERCC6	0.333	0.130	0.381	0.012	0.384
XIRP2	0.318	0.140	0.379	0.011	0.378
DNMT3A	0.307	0.139	0.367	0.014	0.386
BRAF	0.285	0.107	0.393	0.014	0.286
SDK1	0.182	0.076	0.386	0.006	0.334
AATK	0.264	0.122	0.220	0.011	0.236
CDH11	0.207	0.133	0.430		0.414
EPHA7	0.002	0.065	0.198		0.114

Patient 2

	A	B	C	D	E
JAK3	0.221	0.365	0.041	0.123	0.406
GLI2	0.195	0.337	0.087	0.198	0.326
CRTC1	0.207	0.317	0.066	0.158	0.385
CYB5D2	0.206	0.330	0.036	0.121	0.398
KRAS	0.200	0.319	0.033	0.126	0.387
EP300	0.205	0.358	0.023	0.089	0.382
APC	0.187	0.327	0.019	0.105	0.402
MYH11	0.194	0.325	0.021	0.092	0.398
RB1	0.184	0.322	0.035	0.083	0.401
APC	0.189	0.316	0.027	0.098	0.384
KAT6A	0.178	0.329	0.025	0.088	0.389
GUCY1A2	0.177	0.322	0.024	0.097	0.382
RAF1	0.185	0.328	0.006	0.084	0.399
DCLK1	0.170	0.310	0.022	0.080	0.353
ACVR1B	0.207	0.268	0.031	0.106	0.287
SDK1	0.162	0.267	0.022	0.086	0.305
SOX11	0.119	0.270	0.025	0.112	0.255
DNMT3A	0.029	0.010	0.043	0.034	0.005
ERCC4	0.019	0.001	0.004	0.077	0.001
CSMD3		0.185		0.008	0.372
EPHA3		0.196		0.008	0.387
TNFRSF14	0.016	0.005	0.018	0.023	
CIT		0.179			0.303
FBXW7	0.212	0.345			0.403
ADAMTSL3	0.023		0.012		
ROBO2		0.116			
CTTN	0.089				
DCC	0.025				
MAP7		0.024			
PREX2		0.024			

Patient 3

	A	B	C	D	E
AMER1	0.327	0.036	0.093	0.688	0.070
POLE	0.171	0.039	0.131	0.363	0.074
KRAS	0.163	0.042	0.124	0.321	0.055
BRAF	0.170	0.029	0.061	0.355	0.035
ACVR2A	0.163	0.021	0.058	0.354	0.040
ERBB2	0.173	0.026	0.064	0.332	0.034
DNAH1	0.178	0.026	0.064	0.322	0.037
TP53	0.165	0.029	0.056	0.331	0.038
RNF43	0.158	0.018	0.073	0.333	0.033
APC	0.161	0.019	0.050	0.352	0.029
APC	0.153	0.020	0.047	0.338	0.032
NOTCH1	0.151	0.018	0.072	0.260	0.028
GATA3	0.052			0.063	
STK11	0.144		0.001	0.266	
MAP3K1	0.160			0.317	
SMAD2	0.161			0.321	
SPECC1	0.161			0.308	
ERBB4	0.164			0.297	
PREX2			0.056	0.050	0.061
FAT1		0.015		0.048	0.040
TGFBR1		0.015	0.048	0.036	0.026
MED12	0.064				
MED12					0.054
PDGFRA				0.047	
LRIG3			0.045		
BRCA1			0.044		
XIRP2			0.040		
TESK1				0.040	
CRTC1					0.038
ALK	0.036				
ERCC6	0.035				
NEK8					0.028
ARID1B	0.028				
KDM5B				0.026	
DCLK1					0.023
SMG1					0.022

Patient 4

	A	B	C	D	E
TP53	0.689	0.872	0.878	0.842	0.678
SFPQ	0.634	0.660	0.702	0.622	0.629
TIAM1	0.531	0.705	0.613	0.593	0.503
SOX9	0.540	0.592	0.583	0.562	0.488
APC	0.464	0.603	0.596	0.584	0.501
GRM8	0.356	0.370	0.386	0.370	0.313
FAT4	0.301	0.376	0.302	0.322	0.318
WHSC1	0.091	0.401	0.366	0.299	0.327
TRIO	0.298	0.302	0.311	0.302	0.264
KRAS	0.262	0.294	0.346	0.308	0.260
APC	0.267	0.301	0.302	0.299	0.245
ELL	0.200	0.235	0.236	0.232	0.217
GRM8	0.054	0.217	0.255	0.003	0.002
LAMA1	0.00042	0.016	0.000070		0.00021
TGFBR2	0.070	0.015			0.006
TIAM1	0.071	0.171	0.286		0.202
SRGAP3	0.092	0.001			
STL		0.365	0.478		
SMAD3		0.195			
AKAP9		0.132			
BRSK1					0.109
MYO3B			0.098		
JAK3		0.096			
SMAD4	0.048				
EDNRB	0.024				
