# Supplementary material for: Multiregion ultra‐deep sequencing reveals early intermixing and variable levels of intratumoral heterogeneity in colorectal cancer
Source: Mol Oncol. 2016 Oct 20;11(2):124–39. doi: 10.1002/1878-0261.12012 (PMC5527459; doi:10.1002/1878-0261.12012)
Supplement: Supplementary file 2 — Fig. S2. ASCAT profiles of twenty tumor sectors belonging to four patients. [file MOL2-11-124-s002.pdf]

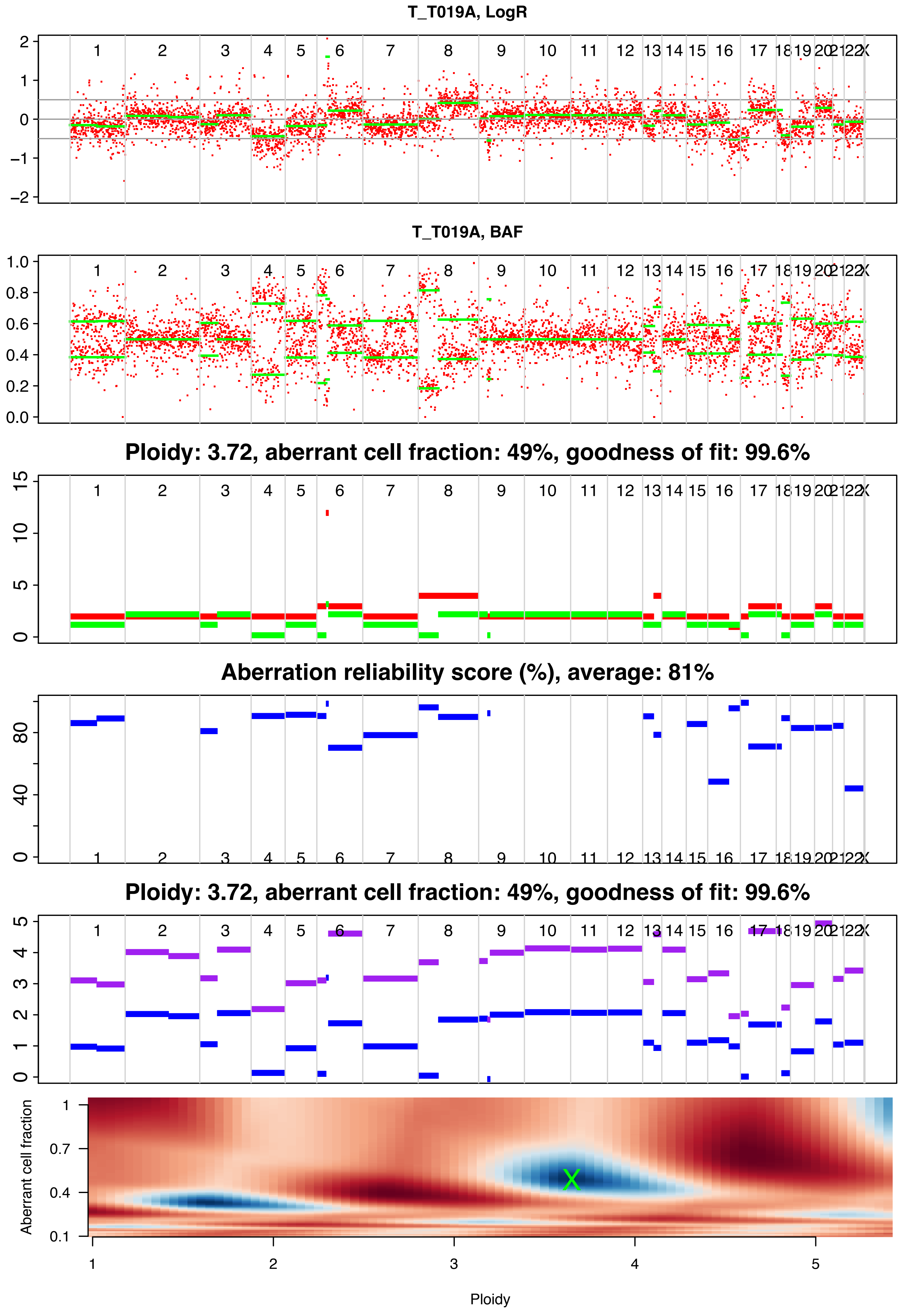
T_T019A, LogR
T_T019A, BAF
Ploidy: 3.72, aberrant cell fraction: 49%, goodness of fit: 99.6%
Aberration reliability score (%), average: 81%
Ploidy: 3.72, aberrant cell fraction: 49%, goodness of fit: 99.6%
Aberrant cell fraction
Ploidy

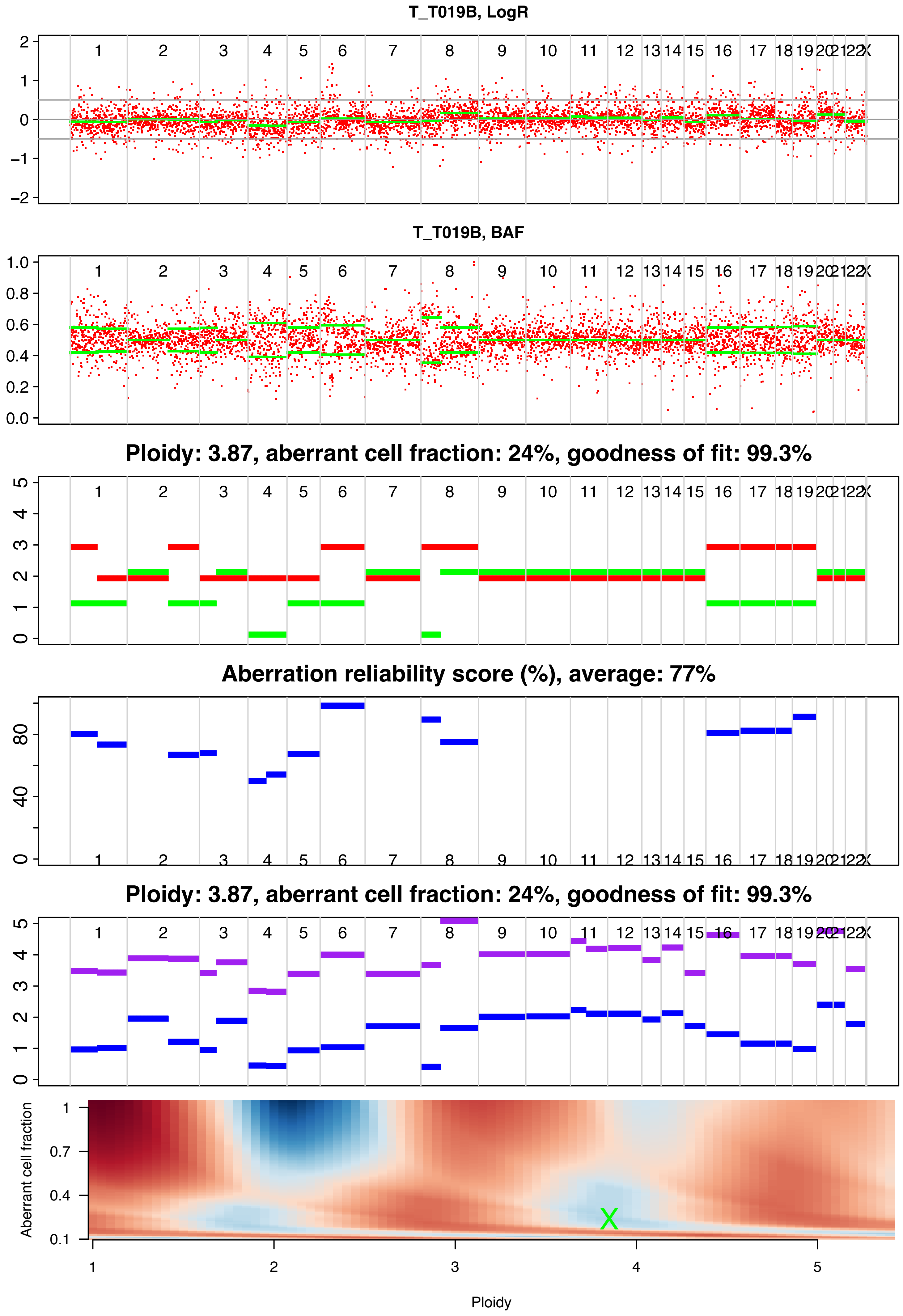
T_T019B, LogR
T_T019B, BAF
Ploidy: 3.87, aberrant cell fraction: 24%, goodness of fit: 99.3%
Aberration reliability score (%), average: 77%
Ploidy: 3.87, aberrant cell fraction: 24%, goodness of fit: 99.3%
Aberrant cell fraction
Ploidy

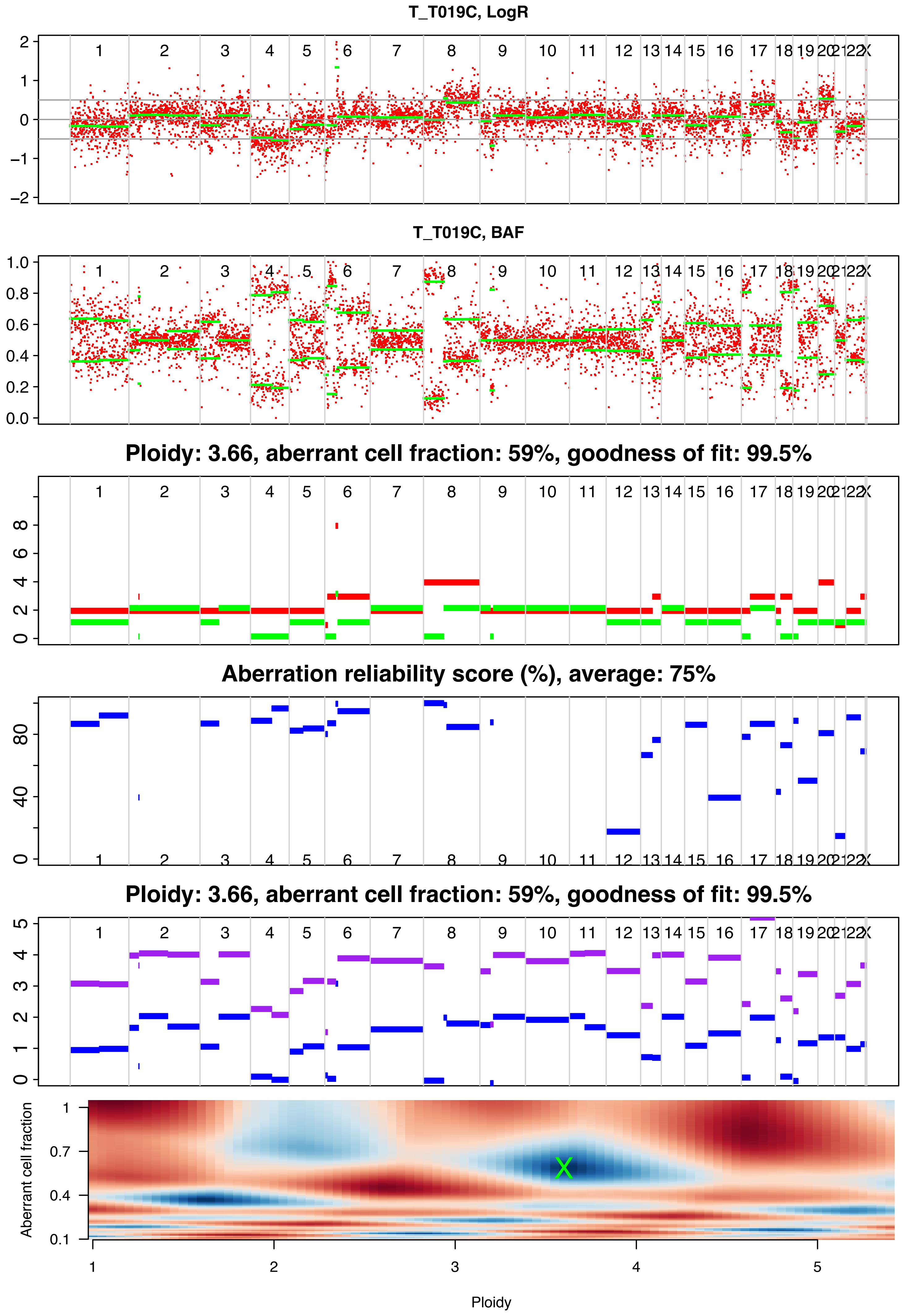
T_T019C, LogR
T_T019C, BAF
Ploidy: 3.66, aberrant cell fraction: 59%, goodness of fit: 99.5%
Aberration reliability score (%), average: 75%
Ploidy: 3.66, aberrant cell fraction: 59%, goodness of fit: 99.5%
Aberrant cell fraction
Ploidy

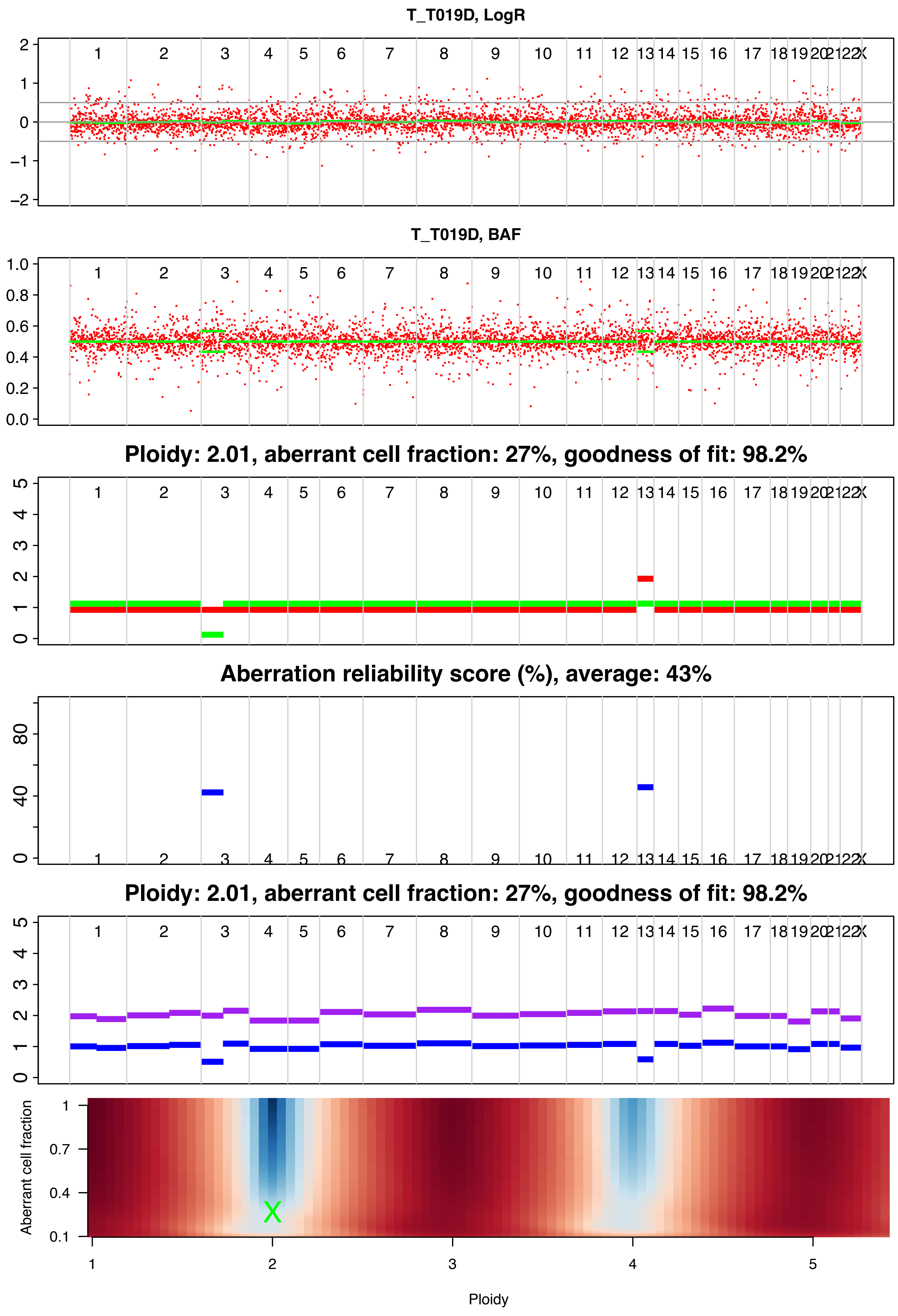

T_T019D, LogR
T_T019D, BAF
Ploidy: 2.01, aberrant cell fraction: 27%, goodness of fit: 98.2%
Aberration reliability score (%), average: 43%
Ploidy: 2.01, aberrant cell fraction: 27%, goodness of fit: 98.2%
Aberrant cell fraction
Ploidy

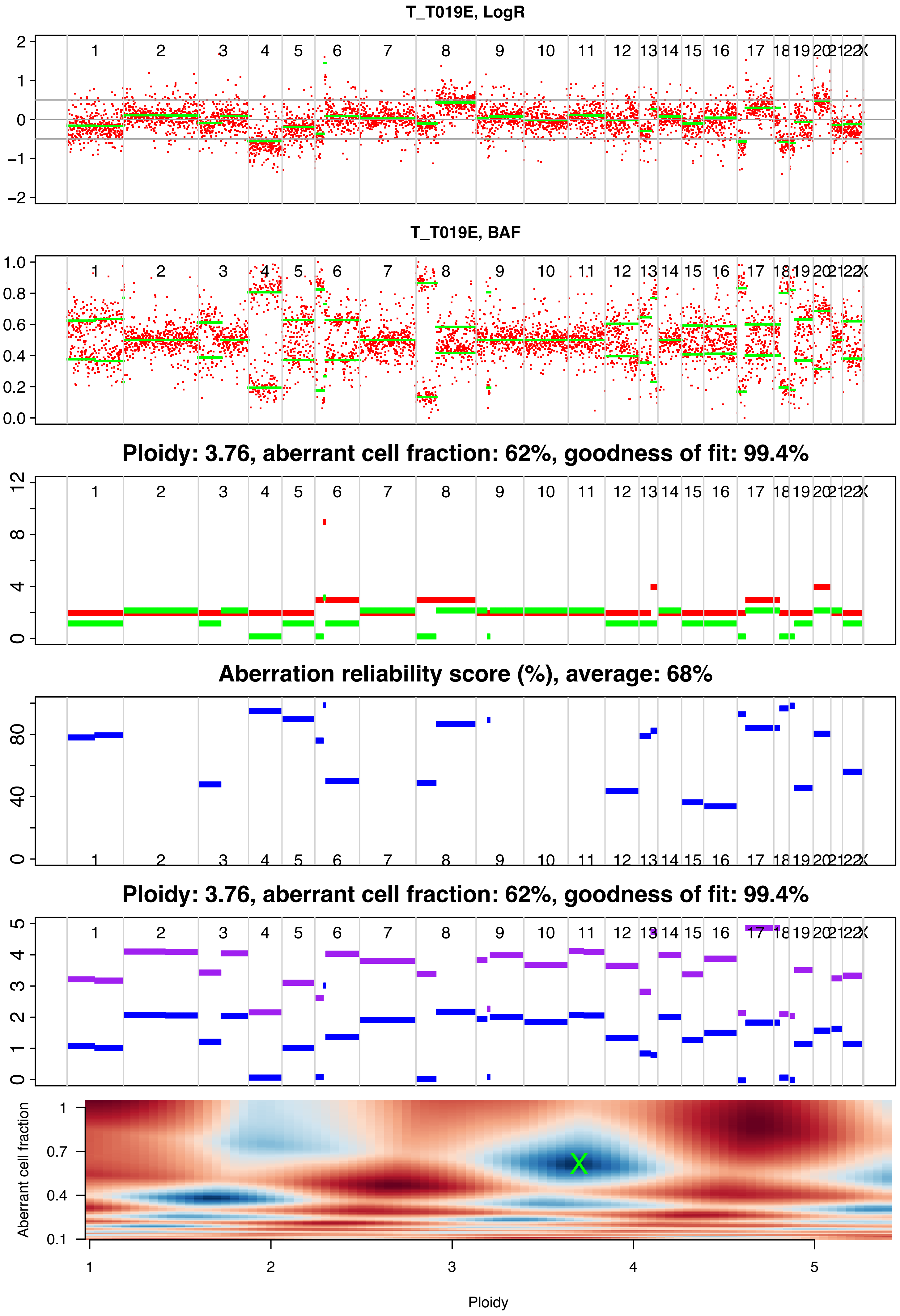
T_T019E, LogR
T_T019E, BAF
Ploidy: 3.76, aberrant cell fraction: 62%, goodness of fit: 99.4%
Aberration reliability score (%), average: 68%
Ploidy: 3.76, aberrant cell fraction: 62%, goodness of fit: 99.4%
1 2 3 4 5 6 7 8 9 10 11 12 13 14 15 16 17 18 19 20 21 22 X
Aberrant cell fraction
Ploidy

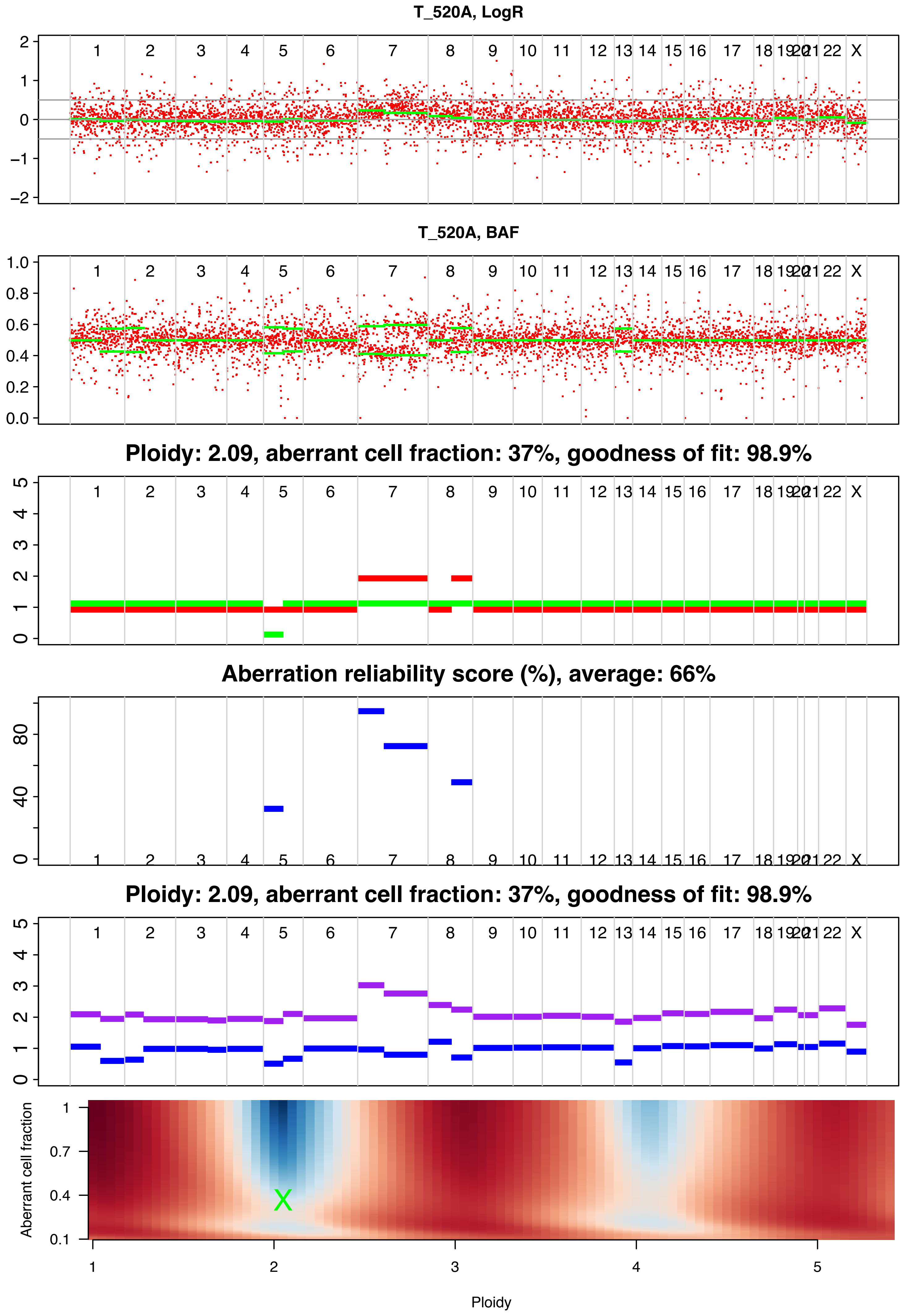
T_520A, LogR
T_520A, BAF
Ploidy: 2.09, aberrant cell fraction: 37%, goodness of fit: 98.9%
Aberration reliability score (%), average: 66%
Ploidy: 2.09, aberrant cell fraction: 37%, goodness of fit: 98.9%
Aberrant cell fraction
Ploidy

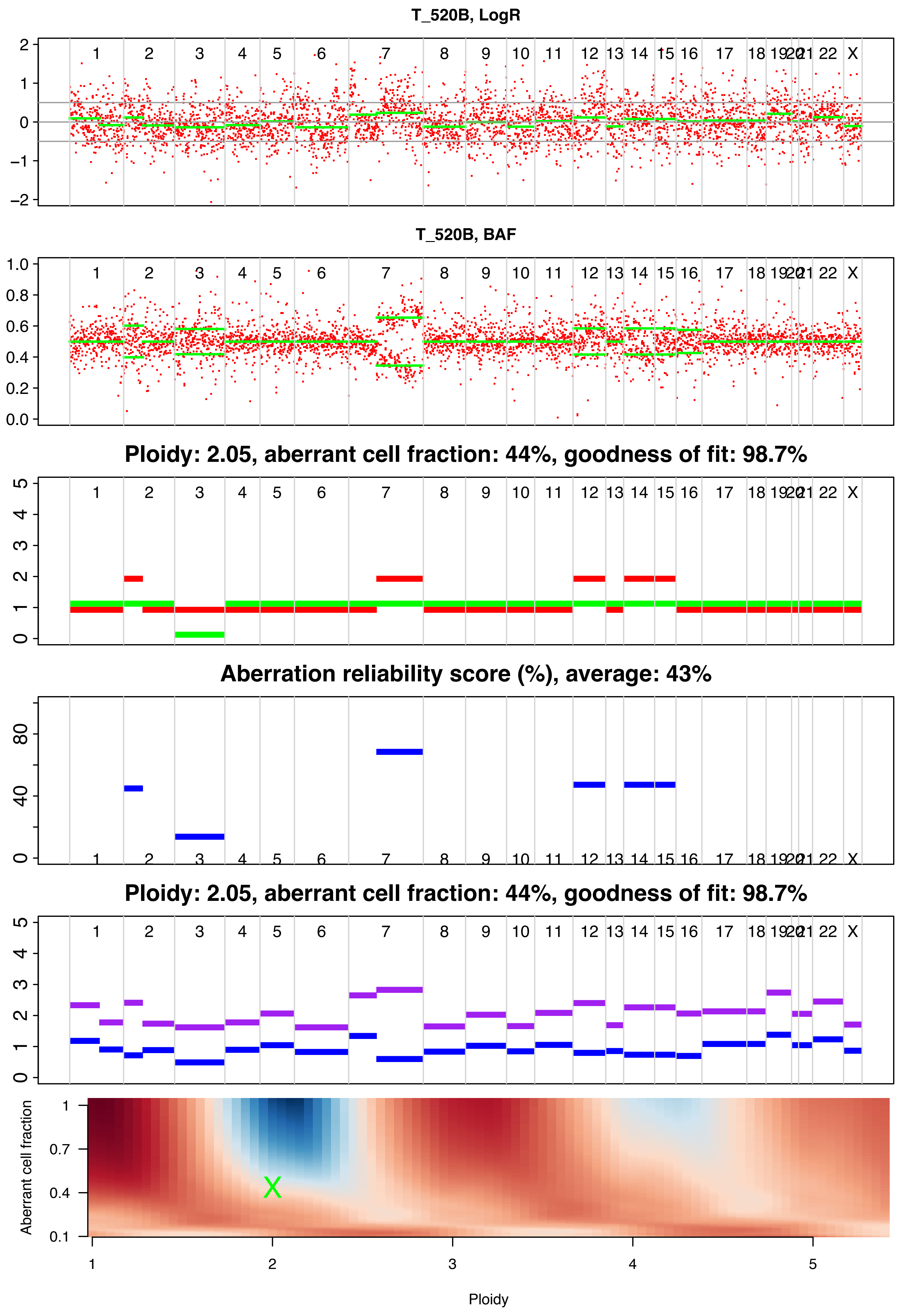
T_520B, LogR
T_520B, BAF
Ploidy: 2.05, aberrant cell fraction: 44%, goodness of fit: 98.7%
Aberration reliability score (%), average: 43%
Ploidy: 2.05, aberrant cell fraction: 44%, goodness of fit: 98.7%
Aberrant cell fraction
Ploidy

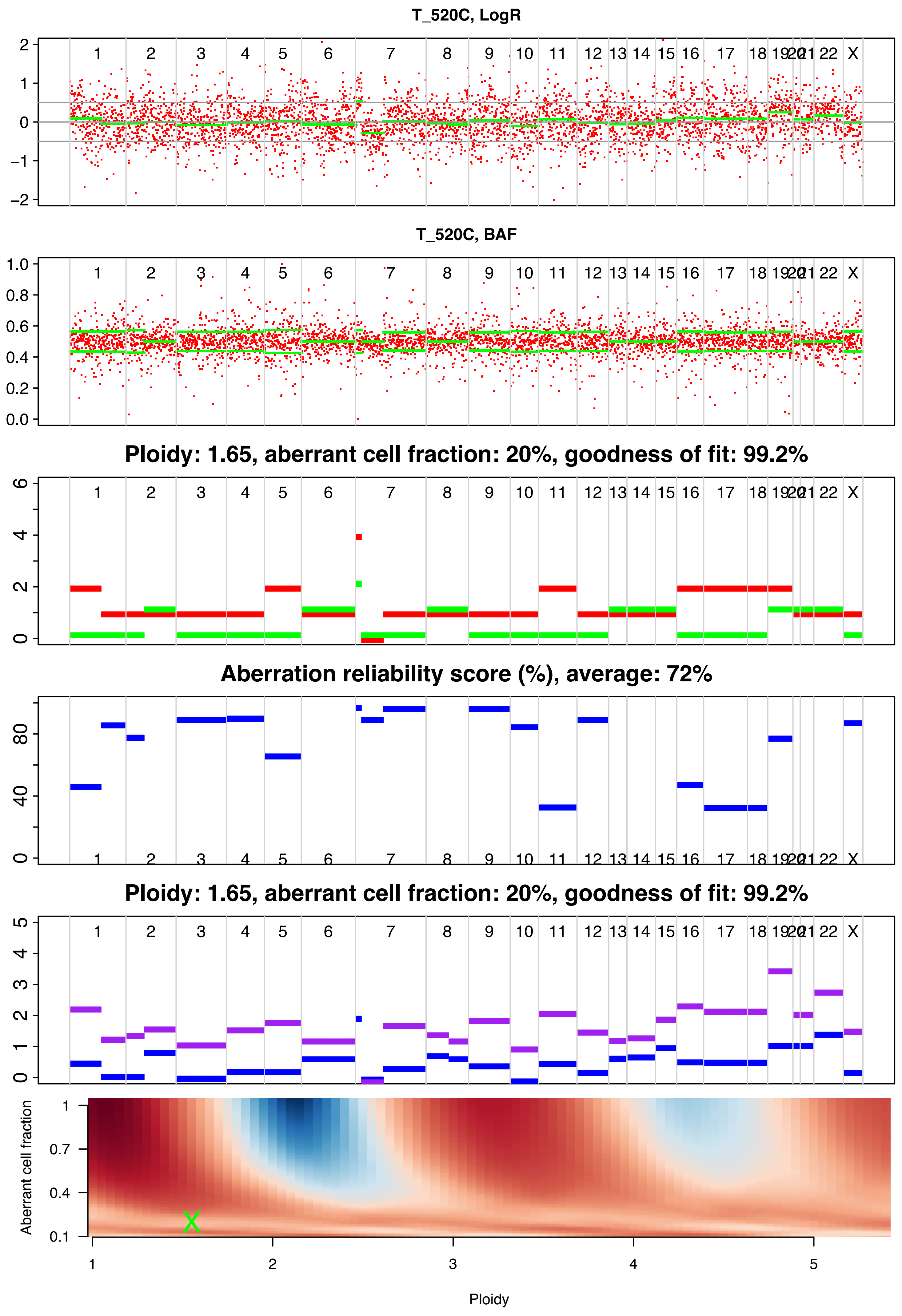
T_520C, LogR
T_520C, BAF
Ploidy: 1.65, aberrant cell fraction: 20%, goodness of fit: 99.2%
Aberration reliability score (%), average: 72%
Ploidy: 1.65, aberrant cell fraction: 20%, goodness of fit: 99.2%
1 2 3 4 5 6 7 8 9 10 11 12 13 14 15 16 17 18 19 20 21 22 X
Aberrant cell fraction
Ploidy

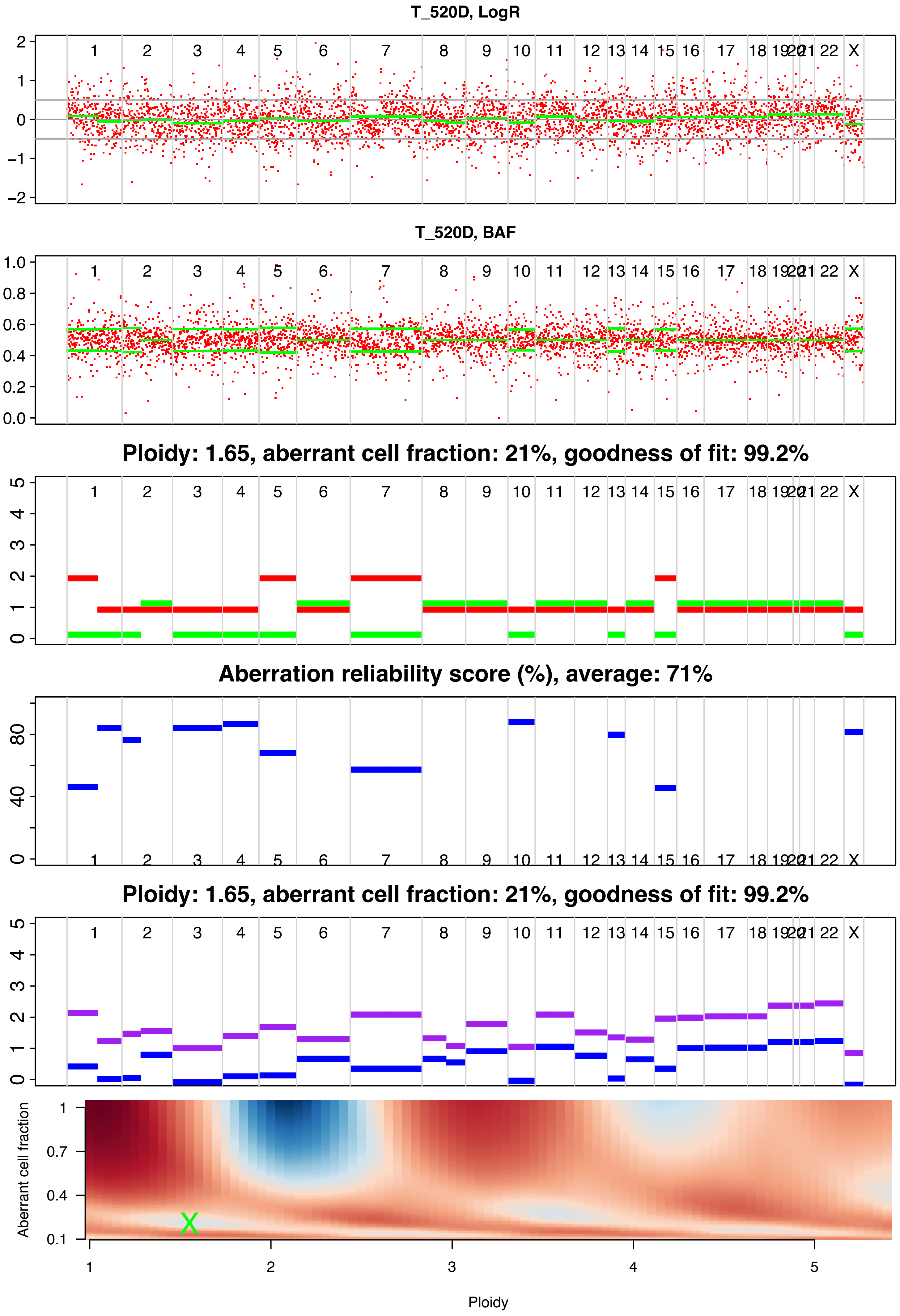

T_520D, LogR
T_520D, BAF
Ploidy: 1.65, aberrant cell fraction: 21%, goodness of fit: 99.2%
Aberration reliability score (%), average: 71%
Ploidy: 1.65, aberrant cell fraction: 21%, goodness of fit: 99.2%
1 2 3 4 5 6 7 8 9 10 11 12 13 14 15 16 17 18 19 20 21 22 X
Aberrant cell fraction
Ploidy

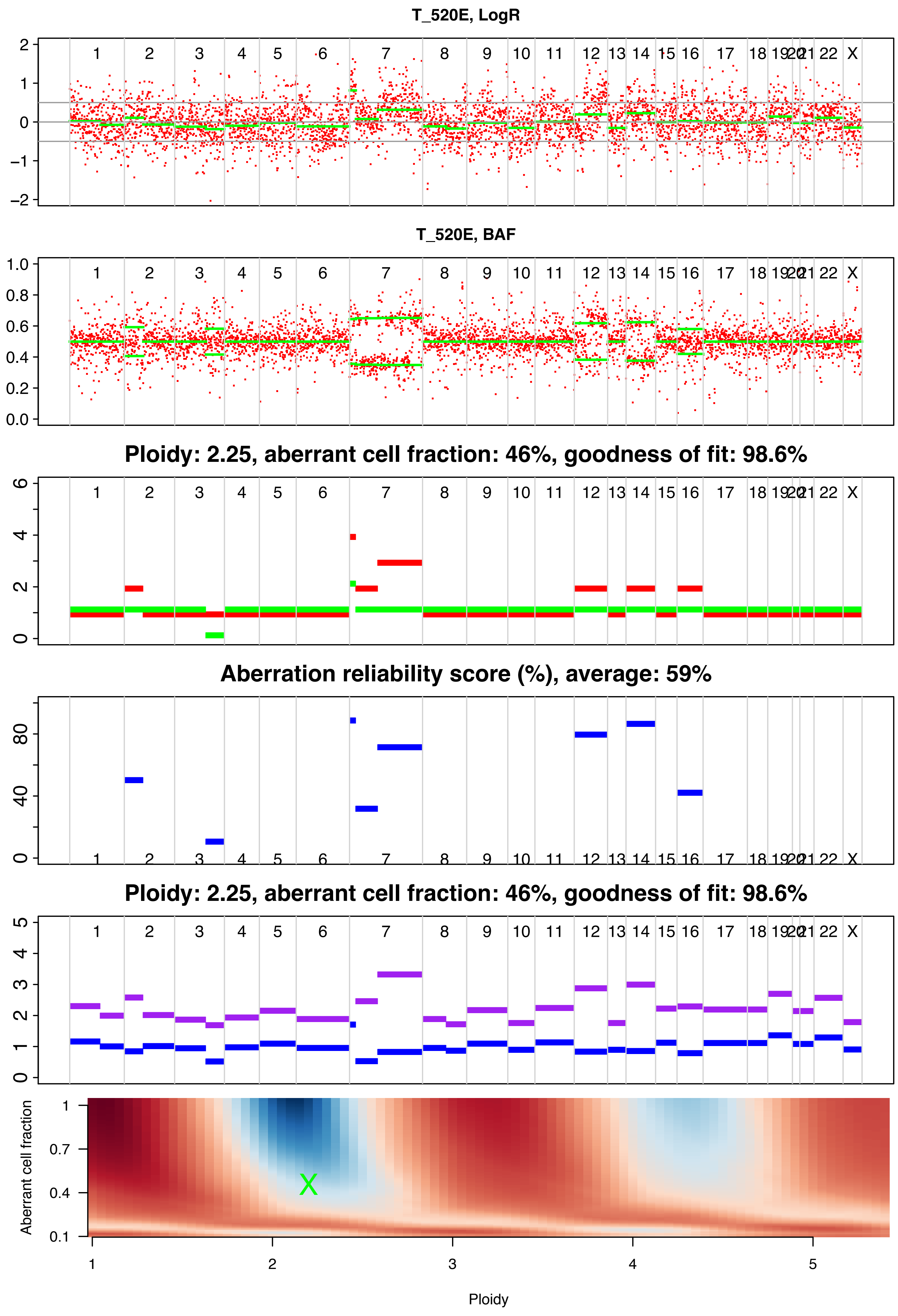
T_520E, LogR
T_520E, BAF
Ploidy: 2.25, aberrant cell fraction: 46%, goodness of fit: 98.6%
Aberration reliability score (%), average: 59%
Ploidy: 2.25, aberrant cell fraction: 46%, goodness of fit: 98.6%
Aberrant cell fraction
Ploidy

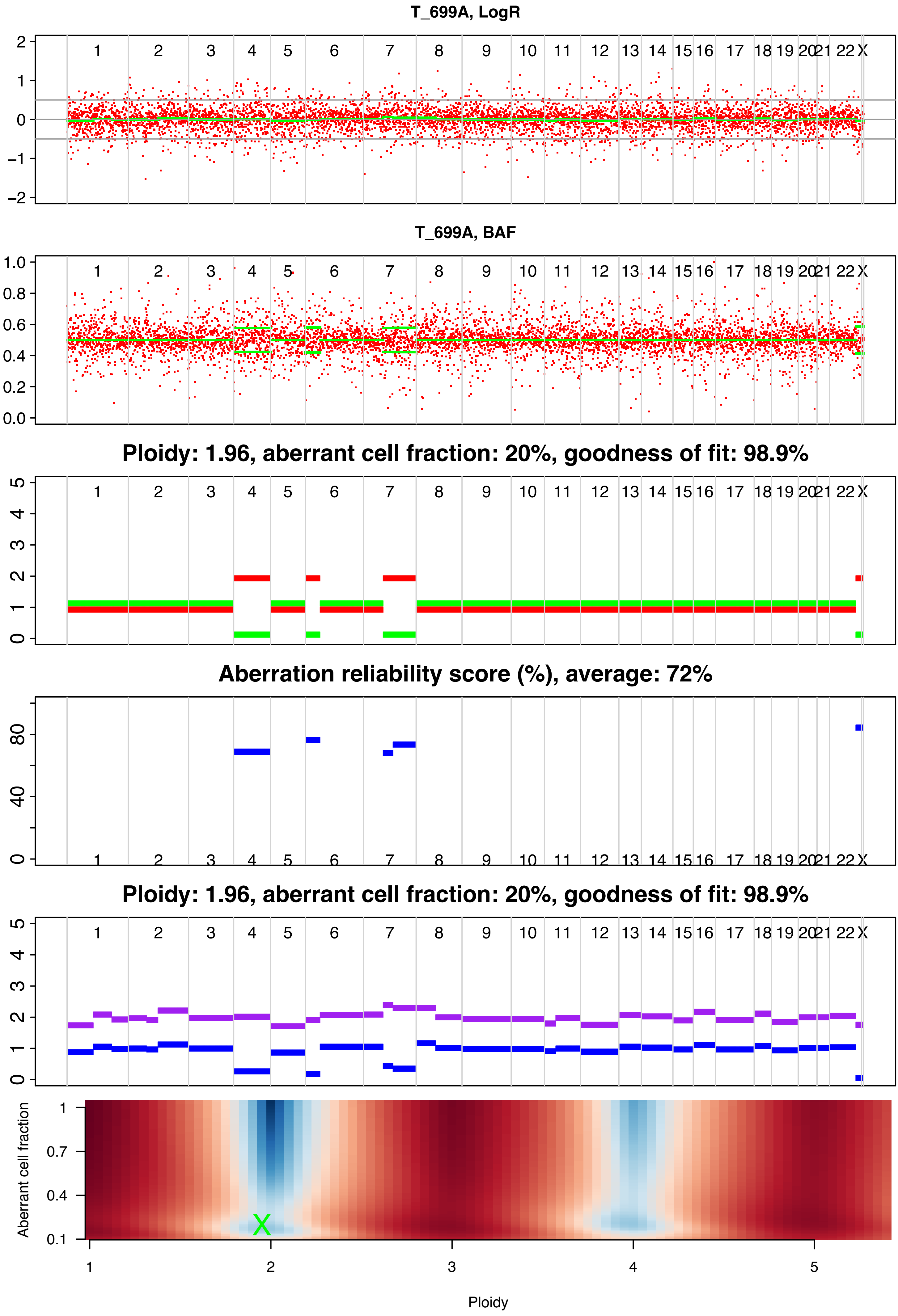

T_699A, LogR
T_699A, BAF
Ploidy: 1.96, aberrant cell fraction: 20%, goodness of fit: 98.9%
Aberration reliability score (%), average: 72%
Ploidy: 1.96, aberrant cell fraction: 20%, goodness of fit: 98.9%
1 2 3 4 5 6 7 8 9 10 11 12 13 14 15 16 17 18 19 20 21 22 X
Aberrant cell fraction
Ploidy

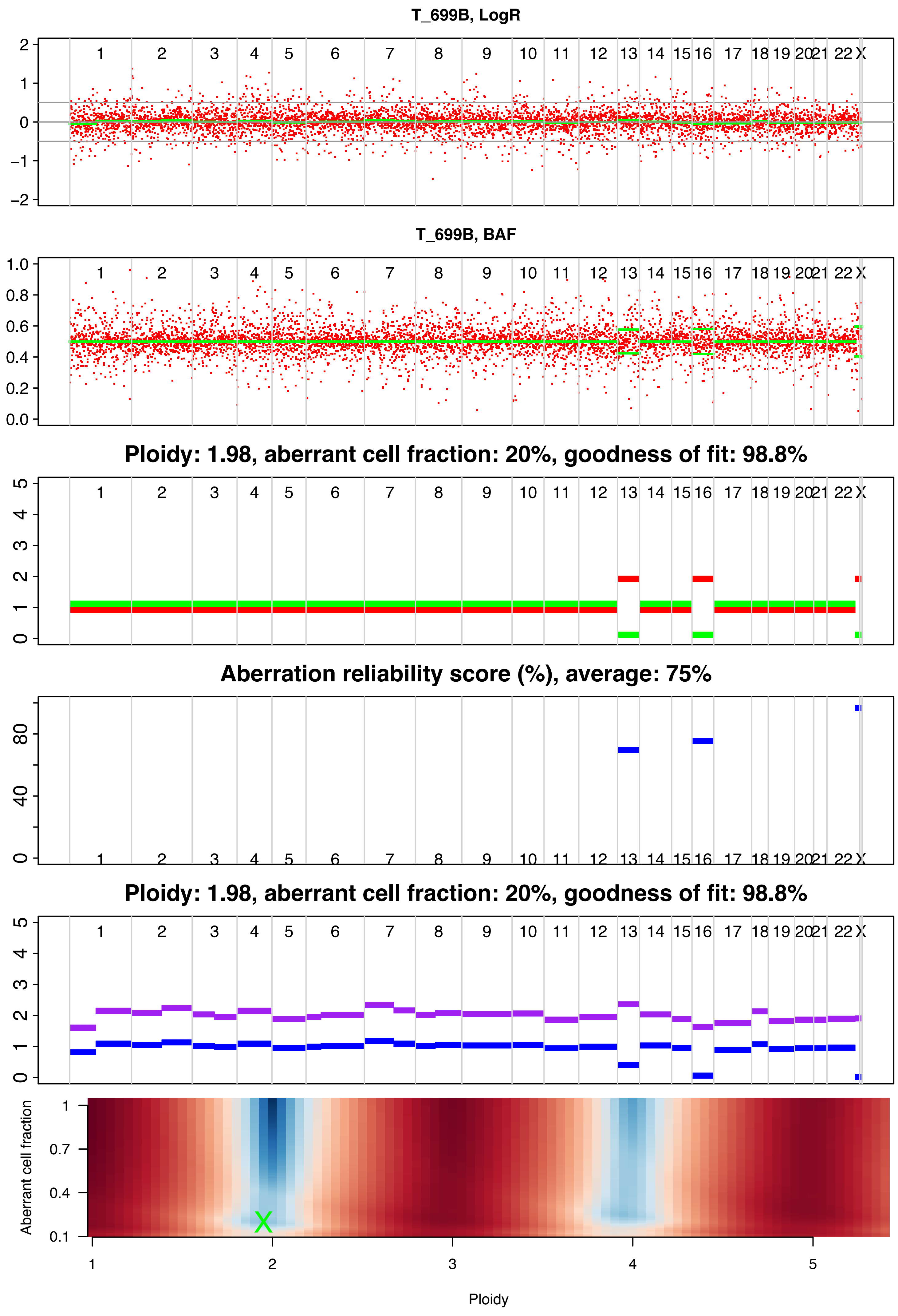
T_699B, LogR
T_699B, BAF
Ploidy: 1.98, aberrant cell fraction: 20%, goodness of fit: 98.8%
Aberration reliability score (%), average: 75%
Ploidy: 1.98, aberrant cell fraction: 20%, goodness of fit: 98.8%
1 2 3 4 5 6 7 8 9 10 11 12 13 14 15 16 17 18 19 20 21 22 X
Aberrant cell fraction
Ploidy

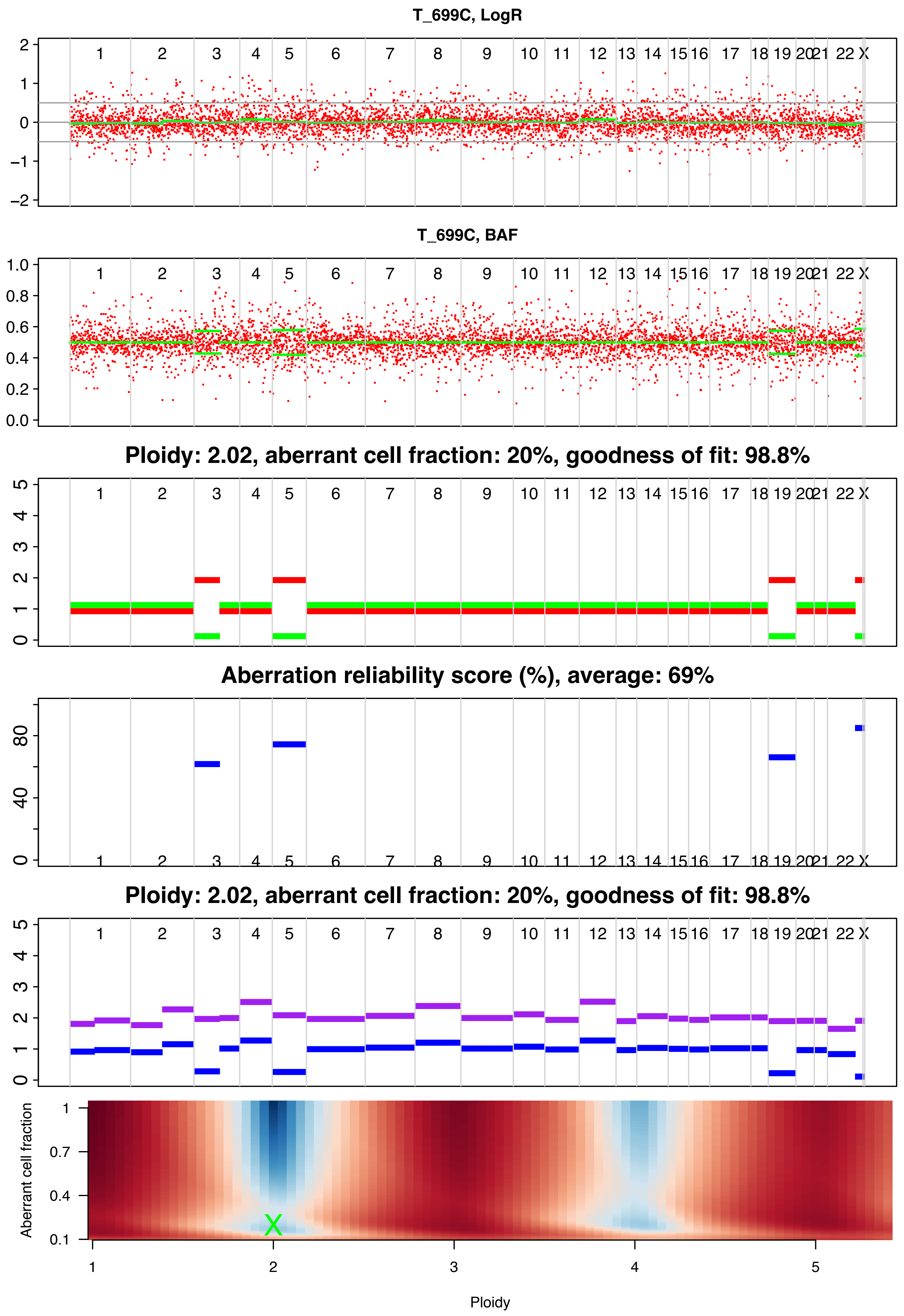

T_699C, LogR
T_699C, BAF
Ploidy: 2.02, aberrant cell fraction: 20%, goodness of fit: 98.8%
Aberration reliability score (%), average: 69%
Ploidy: 2.02, aberrant cell fraction: 20%, goodness of fit: 98.8%
1 2 3 4 5 6 7 8 9 10 11 12 13 14 15 16 17 18 19 20 21 22 X
Aberrant cell fraction
Ploidy

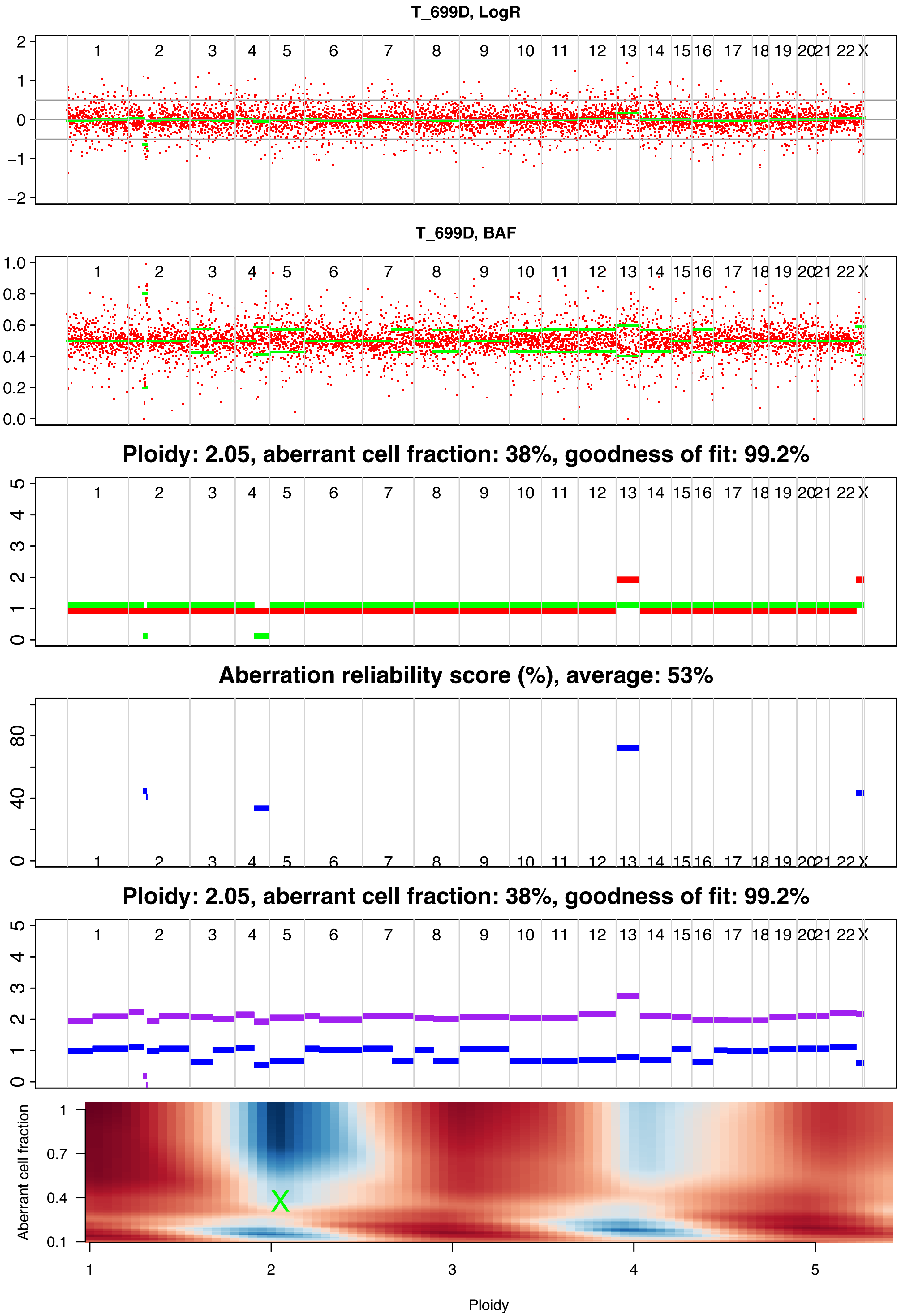

T_699D, LogR
T_699D, BAF
Ploidy: 2.05, aberrant cell fraction: 38%, goodness of fit: 99.2%
Aberration reliability score (%), average: 53%
Ploidy: 2.05, aberrant cell fraction: 38%, goodness of fit: 99.2%
1 2 3 4 5 6 7 8 9 10 11 12 13 14 15 16 17 18 19 20 21 22 X
Aberrant cell fraction
Ploidy

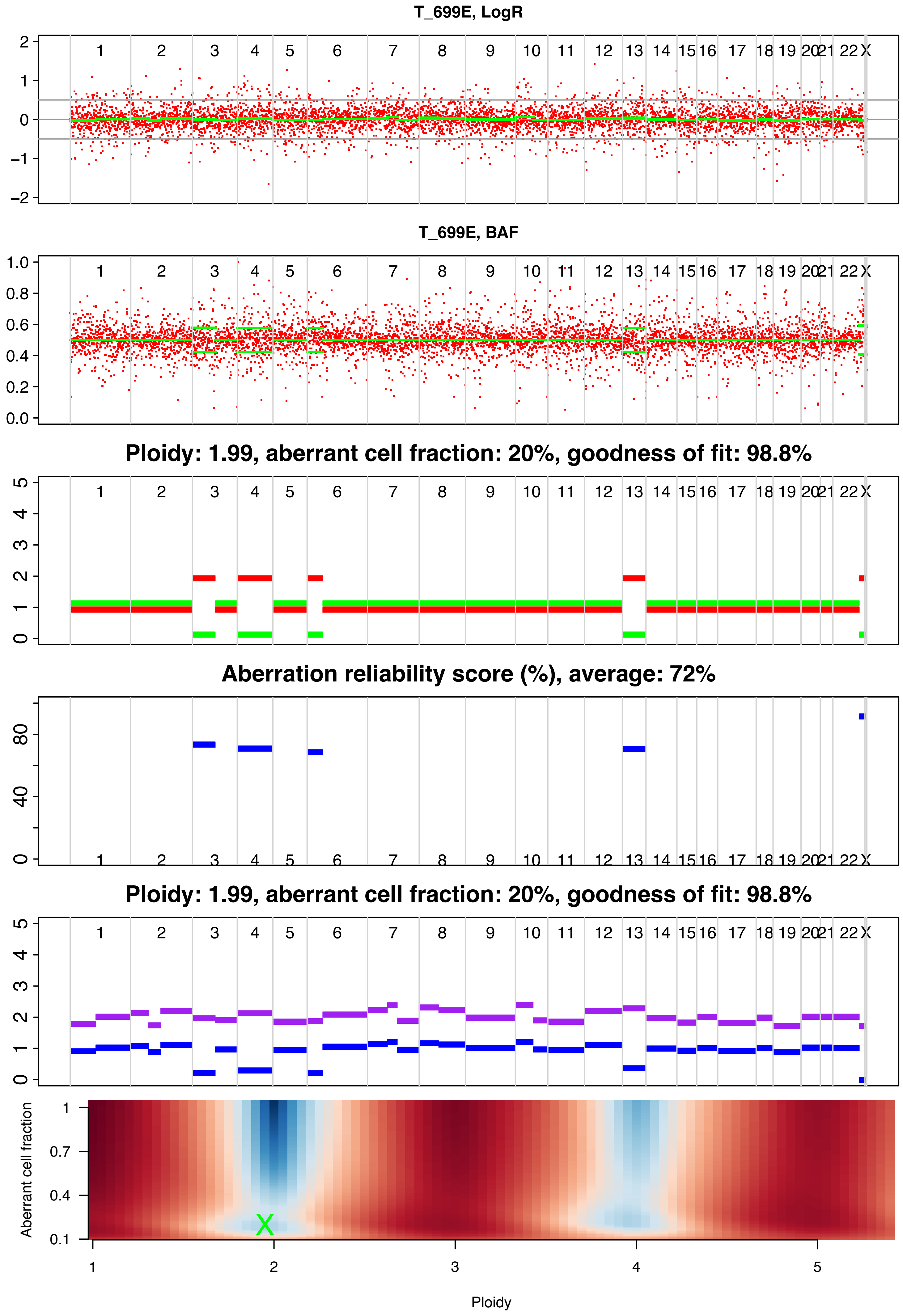

T_699E, LogR
T_699E, BAF
Ploidy: 1.99, aberrant cell fraction: 20%, goodness of fit: 98.8%
Aberration reliability score (%), average: 72%
Ploidy: 1.99, aberrant cell fraction: 20%, goodness of fit: 98.8%
Aberrant cell fraction
Ploidy

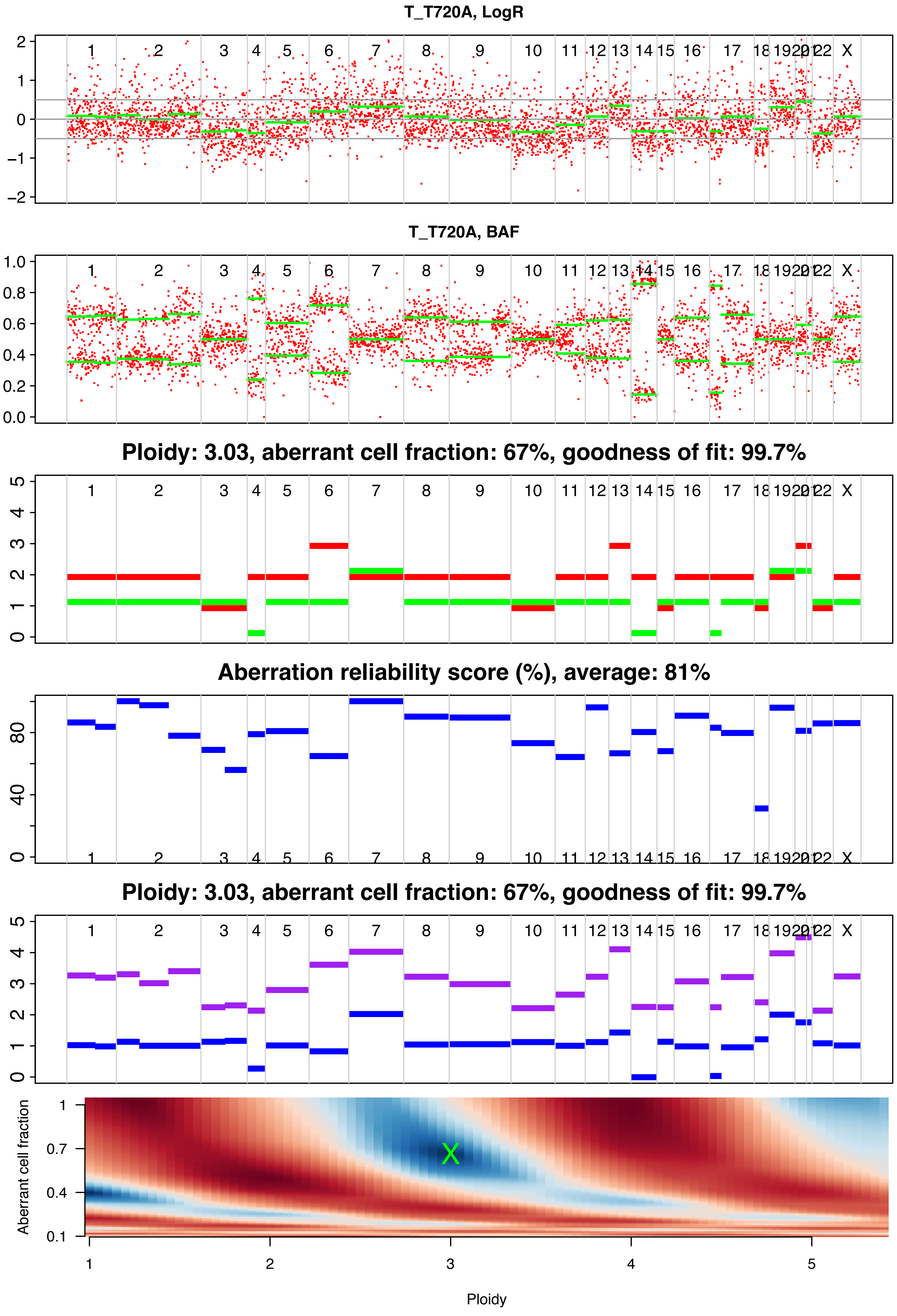
T_T720A, LogR
T_T720A, BAF
Ploidy: 3.03, aberrant cell fraction: 67%, goodness of fit: 99.7%
Aberration reliability score (%), average: 81%
Ploidy: 3.03, aberrant cell fraction: 67%, goodness of fit: 99.7%
Aberrant cell fraction
Ploidy

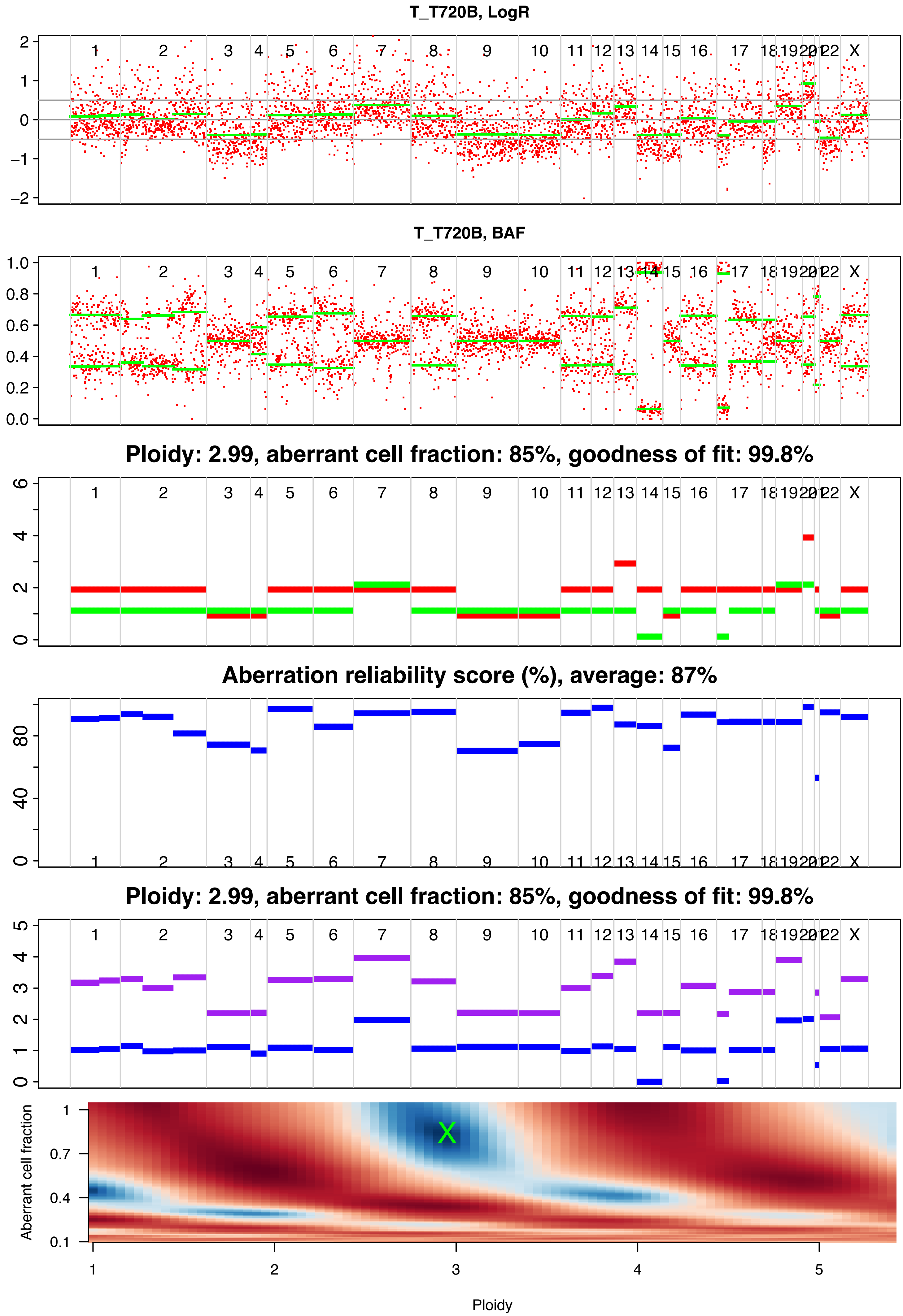
T_T720B, LogR
T_T720B, BAF
Ploidy: 2.99, aberrant cell fraction: 85%, goodness of fit: 99.8%
Aberration reliability score (%), average: 87%
Ploidy: 2.99, aberrant cell fraction: 85%, goodness of fit: 99.8%
Aberrant cell fraction
Ploidy

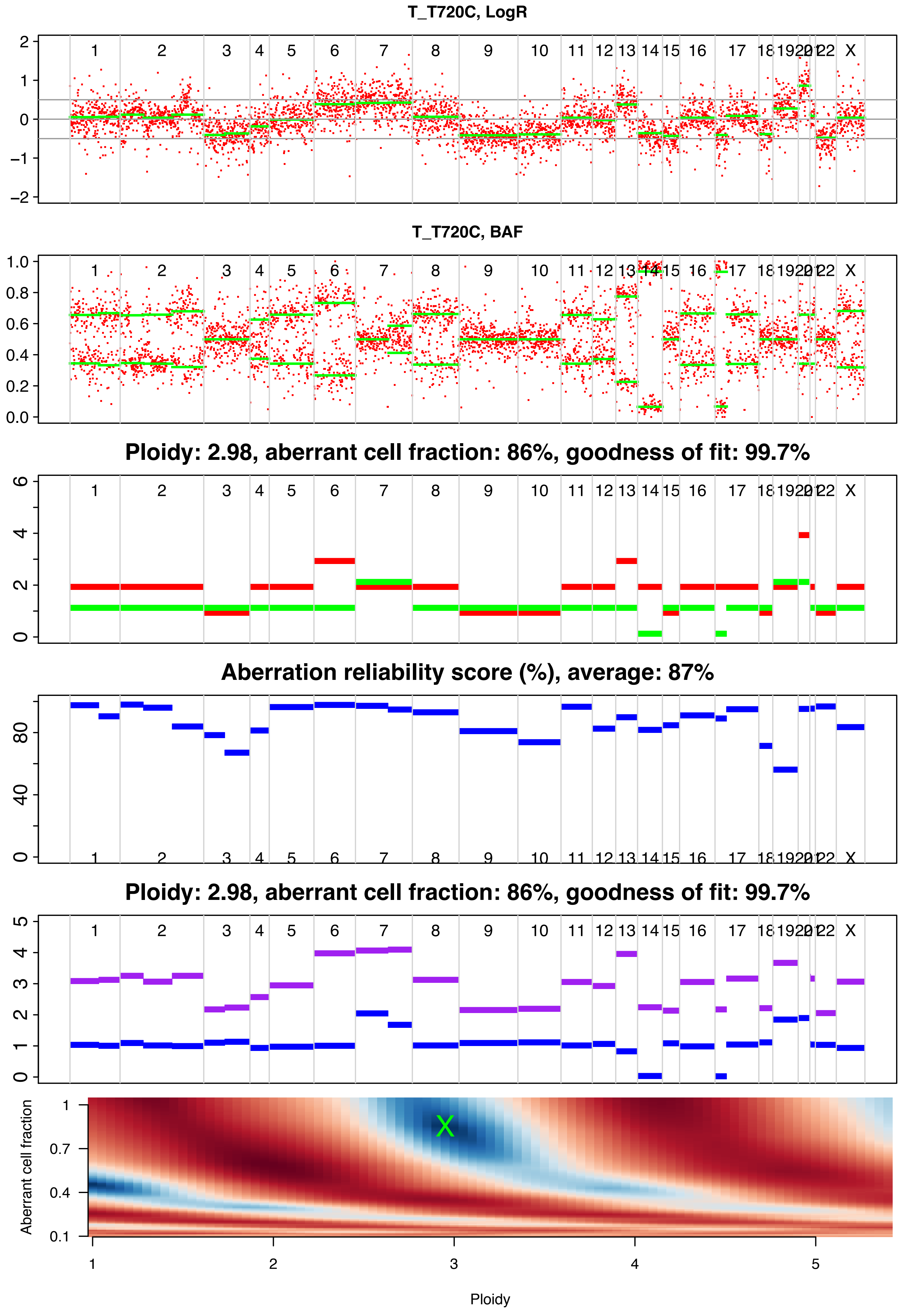

T_T720C, LogR
T_T720C, BAF
Ploidy: 2.98, aberrant cell fraction: 86%, goodness of fit: 99.7%
Aberration reliability score (%), average: 87%
Ploidy: 2.98, aberrant cell fraction: 86%, goodness of fit: 99.7%
Aberrant cell fraction
Ploidy

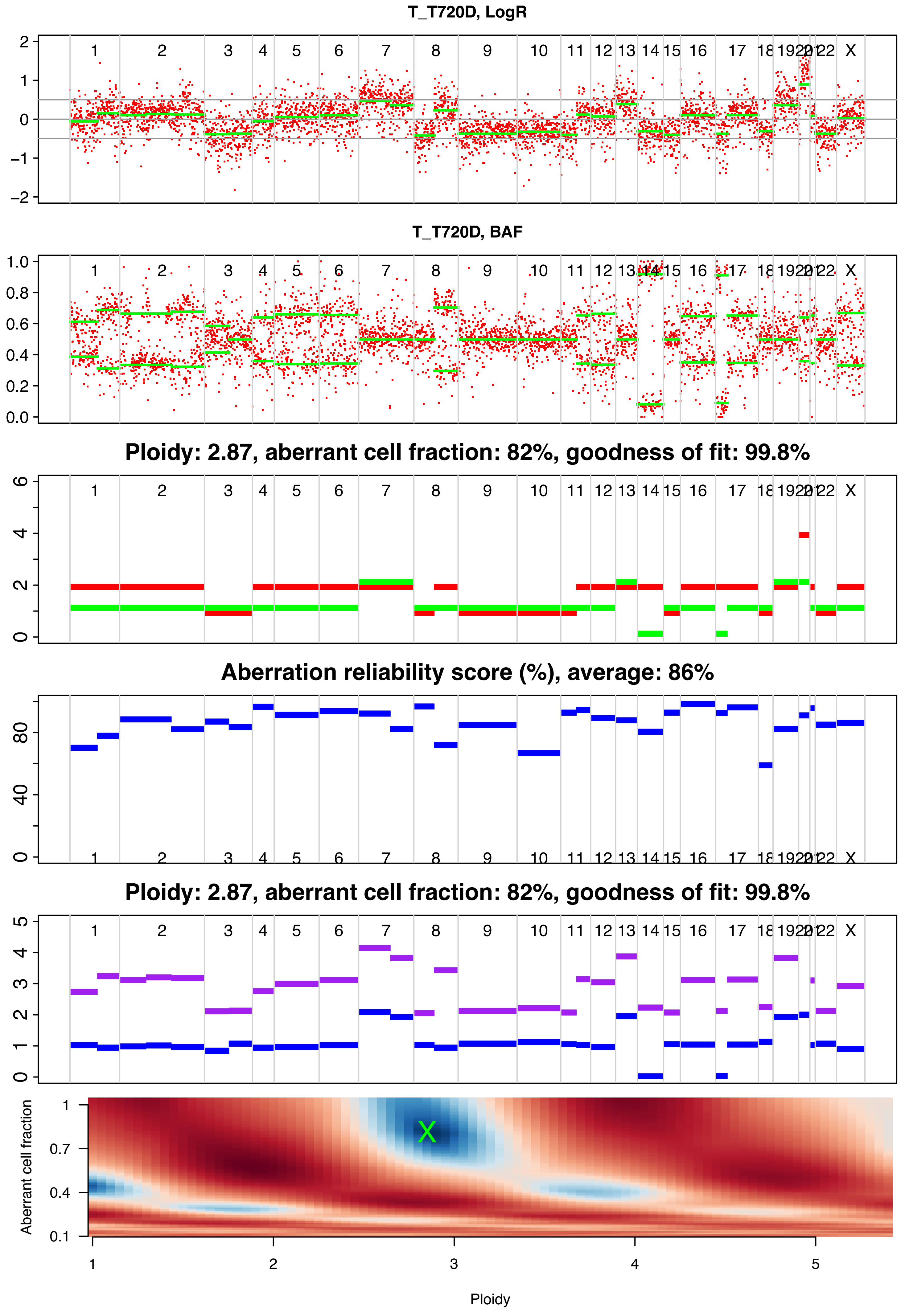
T_T720D, LogR
T_T720D, BAF
Ploidy: 2.87, aberrant cell fraction: 82%, goodness of fit: 99.8%
Aberration reliability score (%), average: 86%
Ploidy: 2.87, aberrant cell fraction: 82%, goodness of fit: 99.8%
Aberrant cell fraction
Ploidy
1 2 3 4 5 6 7 8 9 10 11 12 13 14 15 16 17 18 19 20 21 22 X

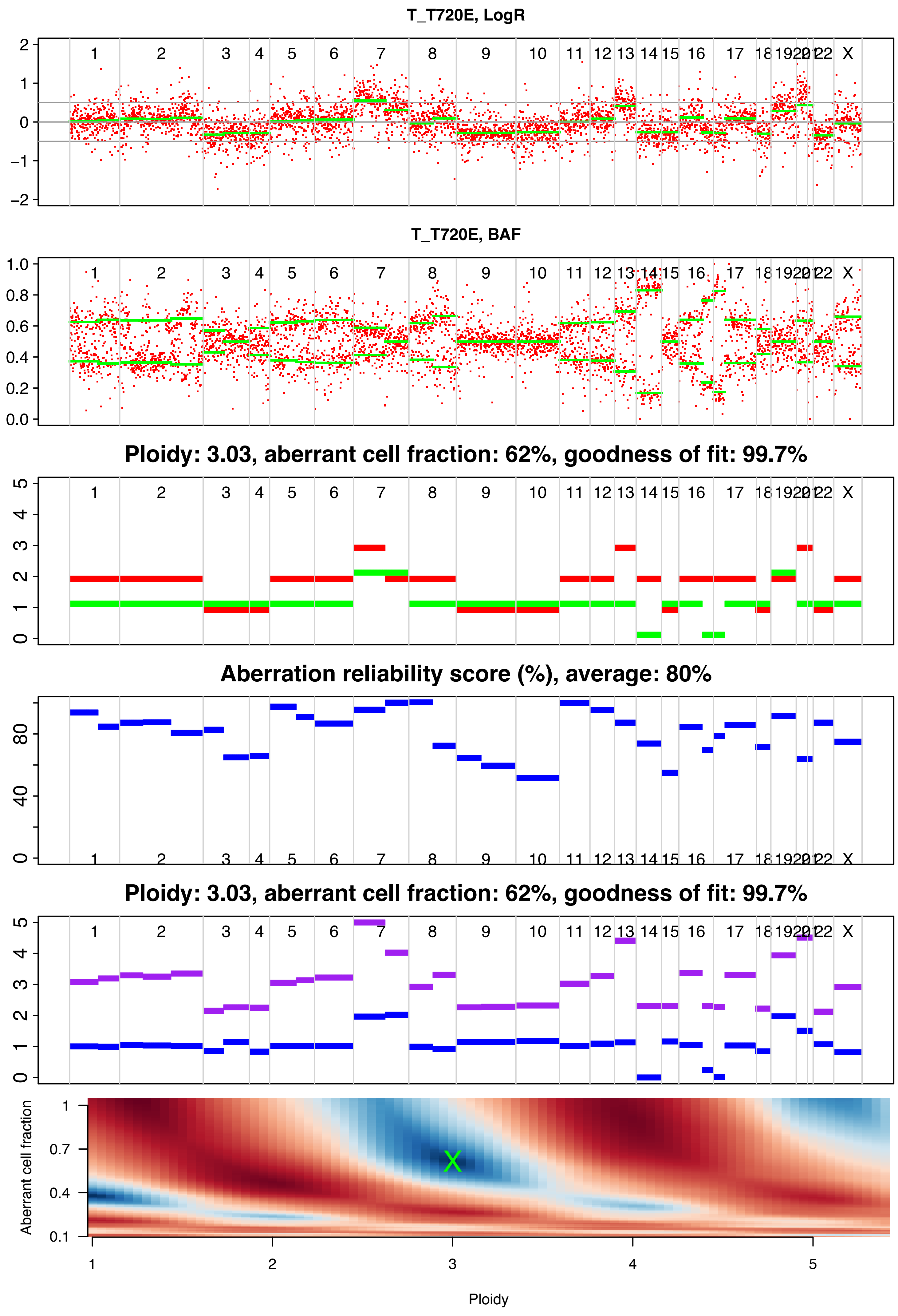

T_T720E, LogR
T_T720E, BAF
Ploidy: 3.03, aberrant cell fraction: 62%, goodness of fit: 99.7%
Aberration reliability score (%), average: 80%
Ploidy: 3.03, aberrant cell fraction: 62%, goodness of fit: 99.7%
Aberrant cell fraction
Ploidy
