# Supplementary figures and images for: Multiregion ultra‐deep sequencing reveals early intermixing and variable levels of intratumoral heterogeneity in colorectal cancer
Source: Mol Oncol. 2016 Oct 20;11(2):124–39. doi: 10.1002/1878-0261.12012 (PMC5527459; doi:10.1002/1878-0261.12012)

ITGB4 P = 0 P.adj = 0.001

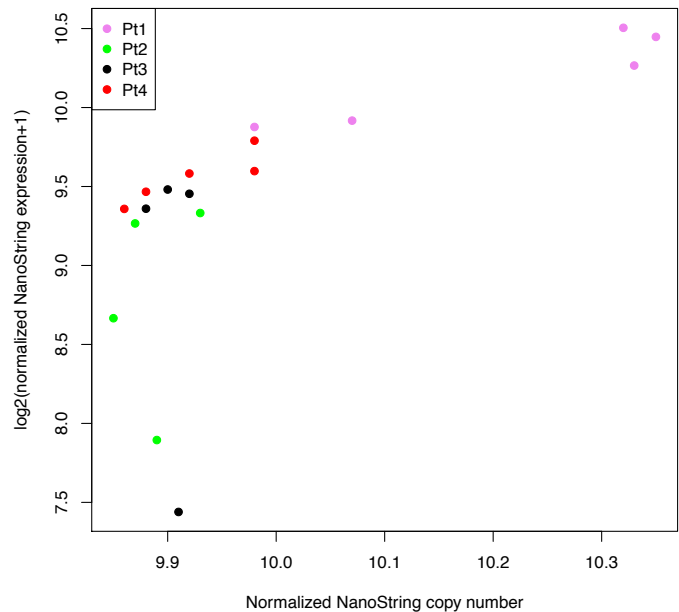

GRB2 P = 0.001 P.adj = 0.008

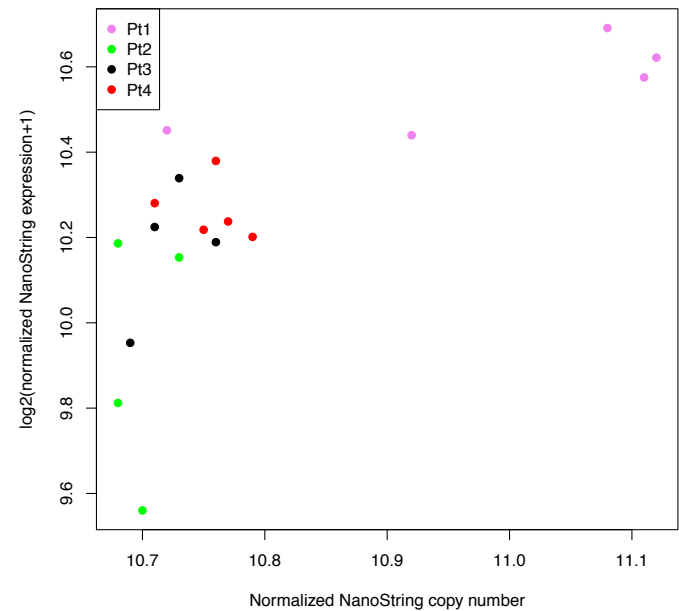

VEGFA P = 0.001 P.adj = 0.011

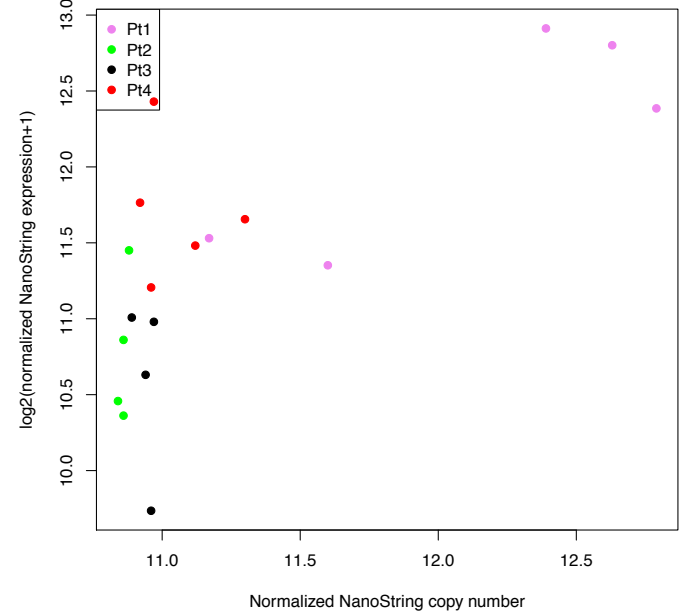

Supplement: Supplementary file 3 — Fig. S3. Correlations between genomic copy number and expression of ITGB4, GRB2 and VEGFA. [file MOL2-11-124-s003.pdf]

AKT2 P = 0 P.adj = 0.003

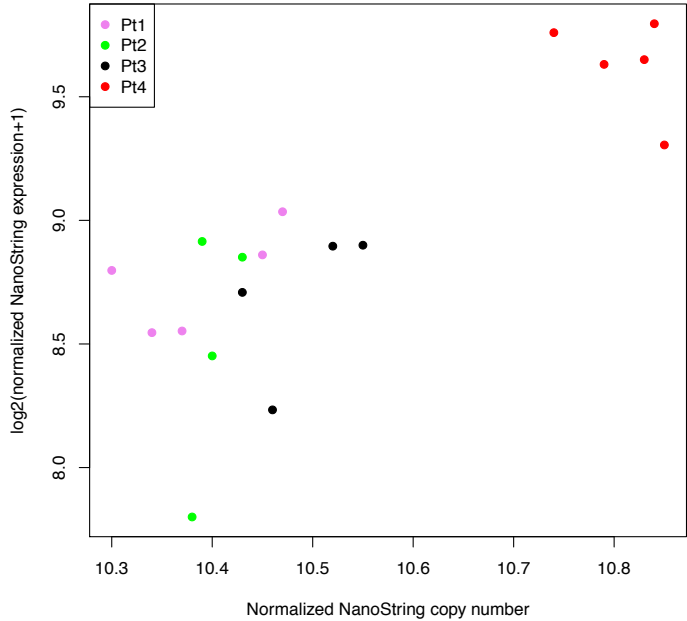

BCL2L1 P = 0.002 P.adj = 0.014

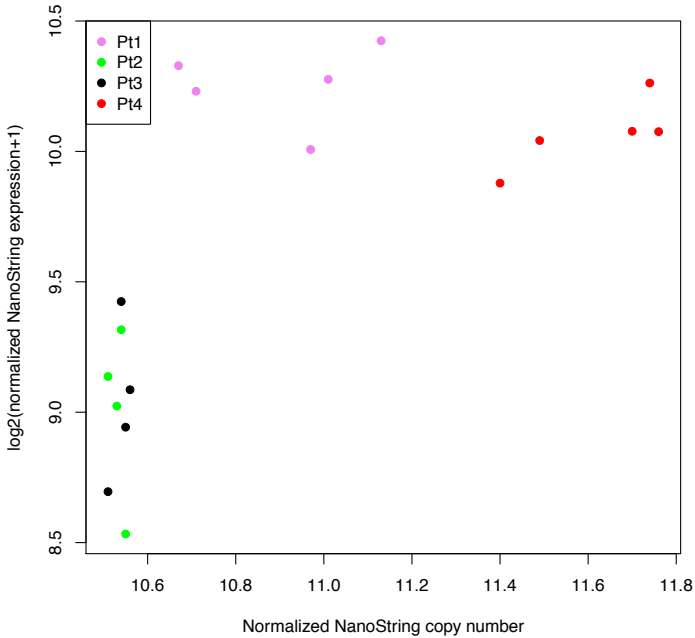

PIK3CA P = 0.016 P.adj = 0.075

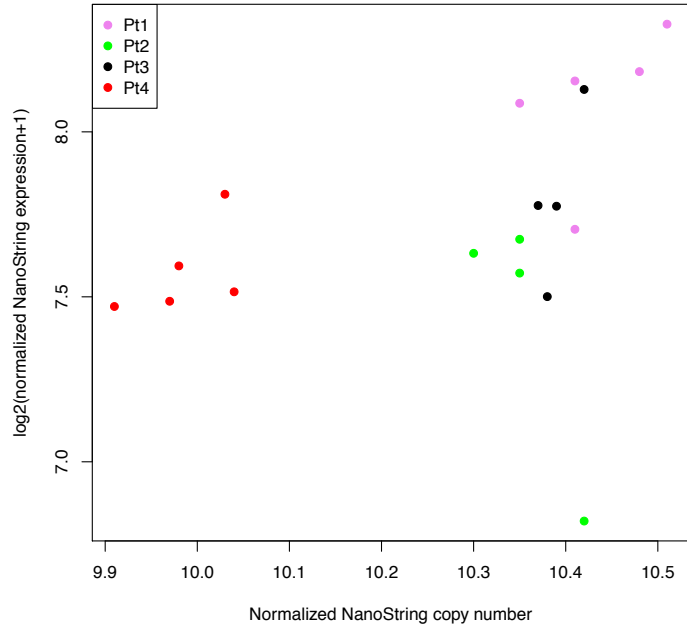

PTEN P = 0.006 P.adj = 0.036

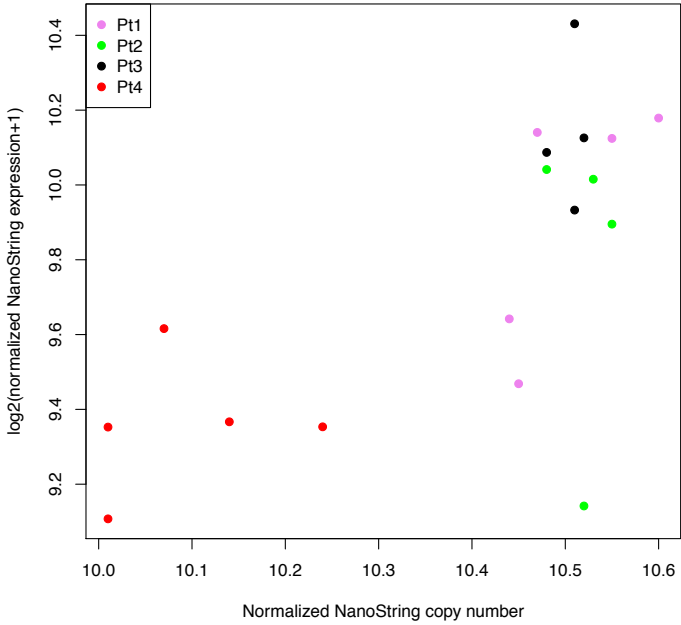

Supplement: Supplementary file 4 — Fig. S4. Elevated copy number and higher expression of AKT2, BCL2L1 and reduced copy number and lower expression of PTEN and PIK3CA. [file MOL2-11-124-s004.pdf]
